# Mapping subnational HIV mortality in six Latin American countries with incomplete vital registration systems

**DOI:** 10.1186/s12916-020-01876-4

**Published:** 2021-01-08

**Authors:** Local Burden of Disease HIV Collaborators

**Affiliations:** Institute for Health Metrics and Evaluation, University of Washington, Seattle, WA USA

**Keywords:** HIV/AIDS, Latin America, HIV mortality, Vital registration, Small area estimation, Mapping, Spatial statistics

## Abstract

**Background:**

Human immunodeficiency virus (HIV) remains a public health priority in Latin America. While the burden of HIV is historically concentrated in urban areas and high-risk groups, subnational estimates that cover multiple countries and years are missing. This paucity is partially due to incomplete vital registration (VR) systems and statistical challenges related to estimating mortality rates in areas with low numbers of HIV deaths. In this analysis, we address this gap and provide novel estimates of the HIV mortality rate and the number of HIV deaths by age group, sex, and municipality in Brazil, Colombia, Costa Rica, Ecuador, Guatemala, and Mexico.

**Methods:**

We performed an ecological study using VR data ranging from 2000 to 2017, dependent on individual country data availability. We modeled HIV mortality using a Bayesian spatially explicit mixed-effects regression model that incorporates prior information on VR completeness. We calibrated our results to the Global Burden of Disease Study 2017.

**Results:**

All countries displayed over a 40-fold difference in HIV mortality between municipalities with the highest and lowest age-standardized HIV mortality rate in the last year of study for men, and over a 20-fold difference for women. Despite decreases in national HIV mortality in all countries—apart from Ecuador—across the period of study, we found broad variation in relative changes in HIV mortality at the municipality level and increasing relative inequality over time in all countries. In all six countries included in this analysis, 50% or more HIV deaths were concentrated in fewer than 10% of municipalities in the latest year of study. In addition, national age patterns reflected shifts in mortality to older age groups—the median age group among decedents ranged from 30 to 45 years of age at the municipality level in Brazil, Colombia, and Mexico in 2017.

**Conclusions:**

Our subnational estimates of HIV mortality revealed significant spatial variation and diverging local trends in HIV mortality over time and by age. This analysis provides a framework for incorporating data and uncertainty from incomplete VR systems and can help guide more geographically precise public health intervention to support HIV-related care and reduce HIV-related deaths.

**Supplementary Information:**

The online version contains supplementary material available at 10.1186/s12916-020-01876-4.

## Background

Human immunodeficiency virus (HIV) continues to be a large contributor to morbidity and mortality across the globe [1, 2]. While the burden of the HIV epidemic is most concentrated in sub-Saharan Africa, HIV remains a public health priority in Latin America. In 2017, the Global Burden of Disease Study (GBD) estimated over 30,000 deaths from HIV/AIDS-related causes in the region [1, 2]. To combat the epidemic, the Joint United Nations Program on HIV/AIDS (UNAIDS) Fast-Track strategy emphasizes the need to reduce HIV-related deaths to less than 500,000 deaths worldwide by 2020—a 75% reduction from deaths in 2010 [3]. UNAIDS further targets a 90% reduction in HIV-related deaths by 2030 [3]. Despite increased access to antiretroviral therapy in many countries in Latin America [3, 4], few countries show substantial reduction in HIV mortality since 2000 [1, 2]. Continued efforts are needed to track progress towards meeting the UNAIDS Fast-Track goals with respect to HIV mortality.

Country-level estimates of HIV mortality in Latin America are available from a variety of sources [1, 5] and estimates of mortality exist at the state level in select countries such as Brazil and Mexico [1]. Beyond this, however, detailed subnational estimates at the second administrative level in many countries in Latin America are absent. This lack of subnational estimates is alarming given the localized burden of HIV in urban areas and among high-risk subgroups such as people who inject drugs, sex workers, and men who have sex with men (MSM) [6–10]. Additionally, inequalities in HIV burden across local geographies likely occur given that many underlying drivers of HIV infection and death—such as poverty, incarceration, undernutrition, distribution of health practitioners, and access to health services—vary across geographic areas and through time [11–14]. Previous studies have confirmed substantial within-country variation in mortality rates but are limited to select countries, areas, and years [15–18].

The paucity of evidence on subnational HIV mortality is likely due to several methodological challenges associated with a granular spatial modeling of HIV mortality in Latin American countries. Deaths attributable to HIV are inherently small in number in areas with small populations, adding stochastic noise to direct estimates [19]. Past approaches have used Bayesian models that apply small area methods that borrow strength across age, time, and space to produce stable estimates of mortality rates in areas with a small number of deaths [15, 20–22]. An additional complication in HIV mortality estimation is that—due to stigma or misdiagnosis—HIV deaths may be misclassified and coded to other underlying causes of death such as tuberculosis, endocrine disorders, meningitis, or encephalitis [23–25]. Moreover, vital registration (VR) systems in many countries in Latin America are incomplete and not all deaths are recorded in official statistics [23, 25–27]. While a variety of methods have been proposed to estimate the completeness of death registration at the country level [28, 29], standard methods rely on stable population and sex pattern assumptions that often do not hold in small subnational areas [29, 30]. To resolve the difficulties associated with small numbers of deaths and VR completeness, Schmertmann and Gonzaga proposed a Bayesian model framework for small area life expectancy estimation in countries with incomplete VR systems [31]. This method incorporates a novel functional form for mortality that is informed by prior distributions for VR completeness coverage based on empirical evidence.

In this analysis, we address these challenges by utilizing comprehensive cause of death assignment and applying a small area estimation framework that incorporates prior information on VR completeness to produce estimates of HIV mortality and deaths due to HIV by age and sex at the municipality level in six countries in Latin America: Brazil, Mexico, Guatemala, Costa Rica, Colombia, and Ecuador. Our modeling approach leverages information from neighboring areas across space and time to produce estimates across all years of available data. These six countries were selected based on availability of VR data, but not all contained the same range of available years: our analysis extends from 2000 to 2017 in Brazil, Colombia, and Mexico; from 2009 to 2017 in Guatemala; from 2004 to 2014 in Ecuador; and from 2014 to 2016 in Costa Rica.

## Methods

### Overview

This analysis complied with the Guidelines for Accurate and Transparent Health Estimates Reporting (GATHER) [32]. Our ecological study estimated the HIV mortality rate and the number of HIV deaths by age group and sex at the municipality level for all years of available VR (Additional file 1: Figure S1). All analyses were carried out at the second administrative unit level, which we refer to as municipalities for convenience unless referencing country-specific results, where we use the appropriate national nomenclature for the administrative subdivision (municipality for Brazil, Colombia, Guatemala, Mexico; canton for Costa Rica and Ecuador). Municipalities were combined as needed to create stable units of analysis over the study period, reducing the total number of areas analyzed in select countries (Table 1; Additional file 1: Table S1). In the results, all presented rates are age-standardized for comparison between countries, unless otherwise stated. We used standard age weights produced by GBD 2017 for age standardization [1].

**Table 1:** Data availability and administrative characteristics by country

Country	Years of available VR data	Name of second administrative subdivision	Total number of second administrative subdivisions	Number of modeled units in this analysis
Brazil	2000–2017	Municipality	5570	5477
Colombia	2000–2017	Municipality	1122	1115
Costa Rica	2014–2016	Canton	81	81
Ecuador	2004–2014	Canton	224	222
Guatemala	2009–2017	Municipality	340	333
Mexico	2000–2017	Municipality	2458	2441

### Data

#### Vital registration data

Vital registration (VR) mortality data consisted of anonymized individual-level records from all deaths reported in each country’s VR system occurring between the years of study (Additional file 1: Table S2). These records were tabulated by municipality of residence, age group (0–4, 5–9, 10–14, …, 75–79, and ≥ 80 years), sex, and the underlying cause of death according to the tenth revision of the International Classification of Diseases (ICD-10) [33]. We standardized VR data using methods developed for the GBD [1]. This process requires all deaths to be attributed to a single underlying cause of death following ICD guidelines and fits within a hierarchy of mutually exclusive and collectively exhaustive causes. Deaths that were coded with ICD-10 codes that could not be an underlying cause of death, as well as deaths that were coded to non-specific causes of death, were redistributed to most detailed causes of death by age, sex, municipality, and year according to a framework developed by Naghavi et al. [34] and updated for GBD 2017 [1]. This includes an HIV correction step that rectifies deaths assigned to comorbidities such as tuberculosis, endocrine disorders, meningitis, or encephalitis that diverge from locations without HIV epidemics [1].

#### Location hierarchy

We created country-specific location hierarchies that list all subnational administrative units for each year in the specified time period and match each corresponding death from the VR system to the municipality level (Additional file 1: Figure S2). For each country, municipalities were geo-matched to shapefiles provided by the Global Administrative Unit Layers (GAUL) [35] (Brazil, Costa Rica, Guatemala) or the Humanitarian Data Exchange [36] (Colombia, Ecuador, Mexico). In all selected countries, municipality boundaries changed over time, reflecting new boundary designations across the years of study (Table 1). Municipalities that underwent a boundary change during the period of the analysis were merged to create a stable unit across the period of observation, and the municipality-level shapefiles were manually edited to match the split hierarchy using ArcMap version 10.6 [37]. Merged units that included multiple municipalities were modeled as one area, and in the results share the same estimates of HIV mortality rate. Details of these shifts are provided in Additional file 1: Table S1.

#### Covariates and population

We included several available covariates to help inform estimates of HIV mortality: population density [38], night-time light brightness [39], urbanicity [40], and travel time to the nearest settlement of more than 50,000 inhabitants [41] (Additional file 1: Table S3). These covariates were selected because they are factors or proxies for factors previously identified in the literature as associated (not necessarily causally) with HIV mortality. Specifically, these four variables were included as measures or proxies for connectedness and urbanicity as HIV historically spread among high-risk groups in urban areas [6, 42] and is typically found to be higher in more urban compared to more rural locations. Each covariate was obtained in a raster format at a 5 × 5-km resolution and required aggregation to the modeled municipality level for inclusion in our modeling framework. This aggregation was done fractionally: raster cells that crossed municipality borders were fractionally allocated to municipalities in proportion to the covered area.

We created age- and sex-specific populations for each municipality unit by aggregating the WorldPop [38] raster to the modified shapefile, utilizing the same fractional aggregation process. The age- and sex-specific populations for municipalities were then scaled to the national population estimates derived from the GBD [1]. To do so, for each country, sex, age group, and year, we defined a population raking factor as the ratio of the GBD population estimate for that same sex, age group, and year to the sum of the WorldPop population for all municipalities within the country, and then multiplied the WorldPop population estimates for each municipality within the country by this raking factor. This resulted in age- and sex-specific population estimates for each municipality which aligned with the GBD national population sizes and structures.

### Statistical model

#### Vital registration completeness

Expanding on previous literature [31], we used a Bayesian framework that bypasses a lack of identifiability between the mortality rate and completeness estimate by incorporating an informed prior on VR completeness. In this analysis, we incorporated information from GBD [1] on subnational (for Brazil and Mexico) and national VR completeness (for remaining countries) as well as geographic patterns in under-5 VR completeness from past analyses [43] to generate priors on municipality-level VR coverage by two age groups (< 15 years and 15+ years) and year (Additional file 1: Figure S3). We selected these two age groups based on the available national VR completeness estimated in GBD and established literature and expert opinion [31, 44]. The supplemental methods outlined in Additional file 1 summarize our process for generating informed priors on VR completeness in greater detail.

We did not model VR completeness for adults if national GBD completeness estimates exceeded 95% in all years of available VR data (Costa Rica and Colombia). Similarly, we did not model under-15 VR completeness if GBD estimates of child completeness were greater than 90% in all years of available VR data (Costa Rica, Guatemala, Mexico). We therefore modeled adult completeness in Ecuador, Guatemala, Mexico, and Brazil, and child completeness in Ecuador, Colombia, and Brazil.

#### Modeling framework

We estimated HIV mortality separately by sex using a small area estimation framework built upon a model developed in prior modeling studies [15, 45]. This Bayesian hierarchical generalized linear model used a Poisson data likelihood to model the number of HIV deaths in a municipality, year, and age group (supplemental methods in Additional file 1). The Poisson distribution was characterized by a parameter that multiplied the mortality rate, and population by municipality and VR completeness by municipality (Colombia, Ecuador, and Guatemala) or state (Brazil and Mexico). Completeness priors added probabilistic information about VR coverage that allowed estimation of mortality rates given counts of registered deaths. We modeled the log of the mortality rate as a linear combination of terms including random effects with conditional autoregressive distributions to smooth over age, year, and municipality. We also included covariates as fixed effects (see supplemental methods in Additional file 1).

Models were estimated separately for each country and sex and fit using the TMB package [46]. One thousand draws were sampled from the approximated posterior distributions of each modeled parameter and used to construct 1000 draws of HIV mortality (*m*_*j*, *t*, *a*_) for each municipality *j*, year *t*, and age group *a*. We calculated point estimates from the mean of these draws, and the lower and upper bounds of the 95% uncertainty interval from the 2.5th and 97.5th percentiles, respectively, for each age, sex, year, and municipality. Municipality-level estimates for each age, year, and sex were aggregated to the state and national level using a population-weighted average. In Brazil and Mexico, estimates were calibrated to GBD at the state level and estimates were calibrated to national HIV mortality estimates for the remaining four countries. To accomplish this, we calculated the ratio of the national- or state-level estimate from GBD to the mean national estimate derived from population-weighting *m*_*j*, *t*, *a*_, and multiplied all draws of *m*_*j*, *t*, *a*_ by this ratio. We generated the number of HIV deaths for each age-sex-year-municipality by multiplying the mean, lower, and upper bounds of our mortality estimates by the corresponding WorldPop population estimate. We quantified the relative inequality as the mortality rate ratios for municipalities in the 90th percentile versus those in the 10th percentile of mortality rate by year. We calculated the absolute inequality as the difference in mortality between municipalities within a country in the 90th percentile and those in the 10th percentile in terms of mortality rate by year. Throughout our analysis, we qualify statements as statistically significant if the posterior probability of that statement exceeds 95%. We completed our analysis using R version 3.6.3 [47].

#### Model assessment

To assess if including VR completeness in our statistical framework improved model estimates, for the five countries where we applied completeness priors to either children under-15 or adults we also fit a model where the completeness term in the statistical model, ${\pi}_{k,t,{a}^{\ast }}$, was removed. We then compared the ratio of annual national HIV mortality in children under 15 and adults from GBD to 1000 draws of national estimates of annual HIV mortality from the standard and completeness models. This ratio, known as the raking factor, is plotted in Additional file 1: Figure S4–S9. A raking factor closer to 1 indicates better alignment with GBD, and inclusion of completeness priors generally resulted in closer alignment with national GBD mortality estimates.

## Results

### Geographic patterns in HIV mortality rate and notable time trends

#### Brazil

**Fig. 1:**
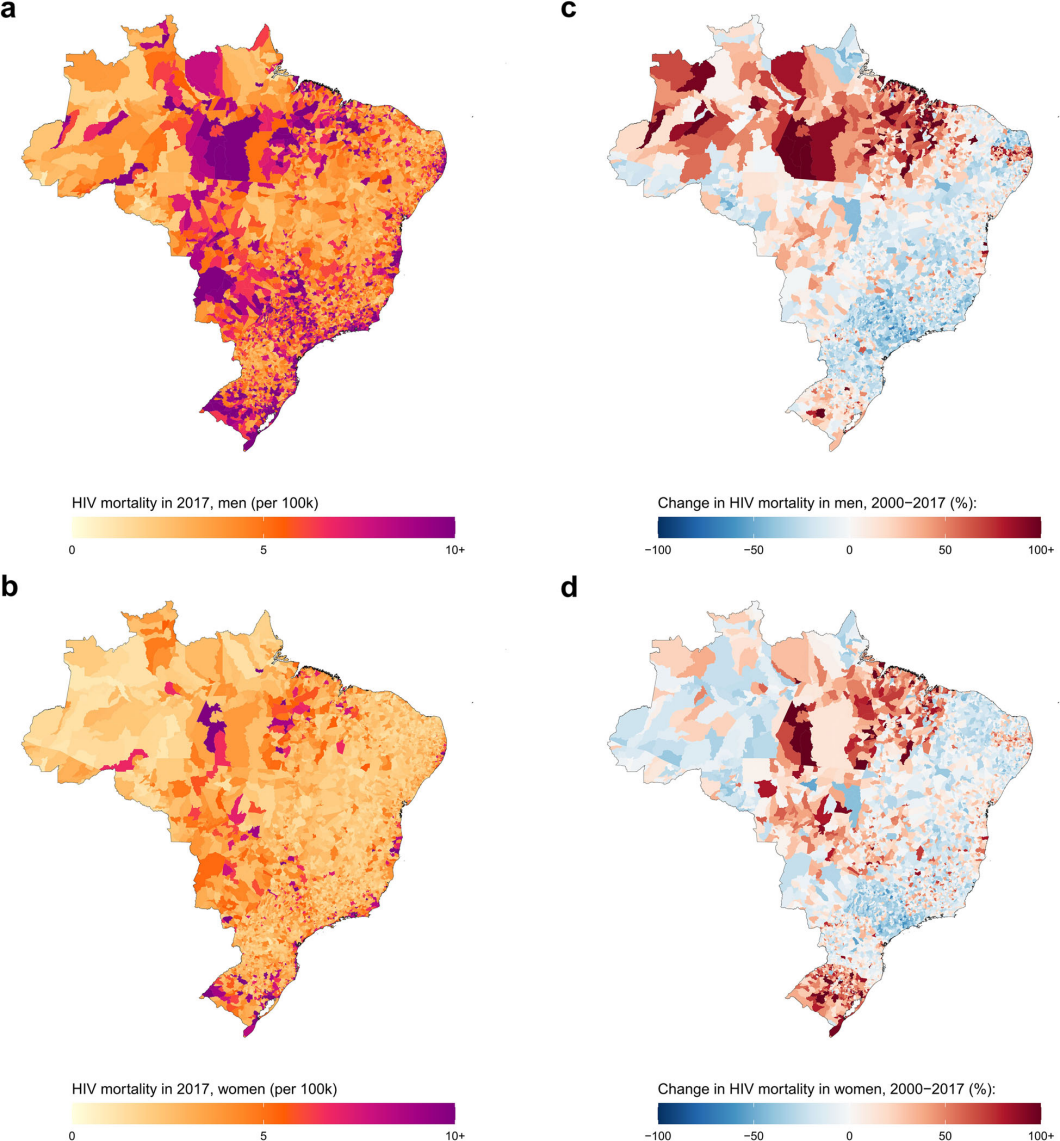
HIV mortality among men and women in Brazil by municipality, 2017. HIV mortality per 100,000 by municipality in Brazil in 2017 among men (**a**) and women (**b**). Relative change in HIV mortality between 2000 and 2017 among men (**c**) and women (**d**)

In Brazil, the estimated national HIV mortality rate for both sexes combined in 2017 was 6.5 (95% uncertainty interval 6.4–6.7) deaths per 100,000, 8.7 [8.5–8.9] deaths among men, and 4.5 [4.4–4.6] deaths among women (Additional file 1: Table S4). Estimated HIV mortality for men in 2017 varied over 53-fold among municipalities: from 0.9 (0.2–2.9) deaths per 100,000 in the Jordão municipality, Acre state to 47.8 (36.4–61.3) deaths per 100,000 in the Tramandaí municipality, Rio Grande do Sul state (Fig. 1 and Additional file 1: Figure S10). Estimated female HIV mortality in 2017 ranged from 0.8 (0.3–1.7) deaths per 100,000 in the Maraã municipality, Amazonas state to 28.6 (20.7–37.7) deaths per 100,000 in the Tramandaí municipality, Rio Grande do Sul state. Between 2000 and 2017, estimated national HIV mortality decreased by 25.3% (from 11.7 [11.4–11.9] deaths per 100,000 in 2000) among men and 14.9% (from 5.3 [5.1–5.4] deaths per 100,000 in 2000) among women. These national decreases hide substantial variation at the municipality level. Estimated male HIV mortality decreased in 3389 (60.8%) municipalities, and 91 [1.6%] municipalities had a statistically significant decrease in HIV mortality. Estimated female HIV mortality decreased in 3000 (53.9%) municipalities and 37 [0.7%] municipalities had a statistically significant decrease in HIV mortality. Changes in estimated male HIV mortality at the municipality level ranged from a 248.1% increase in Bacabal municipality, Maranhão state (from 7.2 [4.9–10.5] deaths per 100,000 in 2000 to 25.1 [18.9–33.2] deaths per 100,000 in 2017) to a 70.9% decrease in Ribeirao Preto municipality, São Paulo state (from 32.6 [28.6–36.9] deaths per 100,000 in 2000 to 9.5 [8.1–11.2] deaths per 100,000 in 2017). Changes in estimated HIV mortality among women at the municipality level varied from a 233.9% increase in Novo Hamburgo municipality, Rio Grande do Sul state (from 5.2 [3.9–6.9] deaths per 100,000 in 2000 to 17.4 [13.8–21.7] deaths per 100,000 in 2017) to a 68.8% decrease in Jundiaí municipality, São Paulo state (from 5.7 [4.4–7.4] deaths per 100,000 in 2000 to 1.8 [1.2–2.5] deaths per 100,000 in 2017).

#### Colombia

**Fig. 2:**
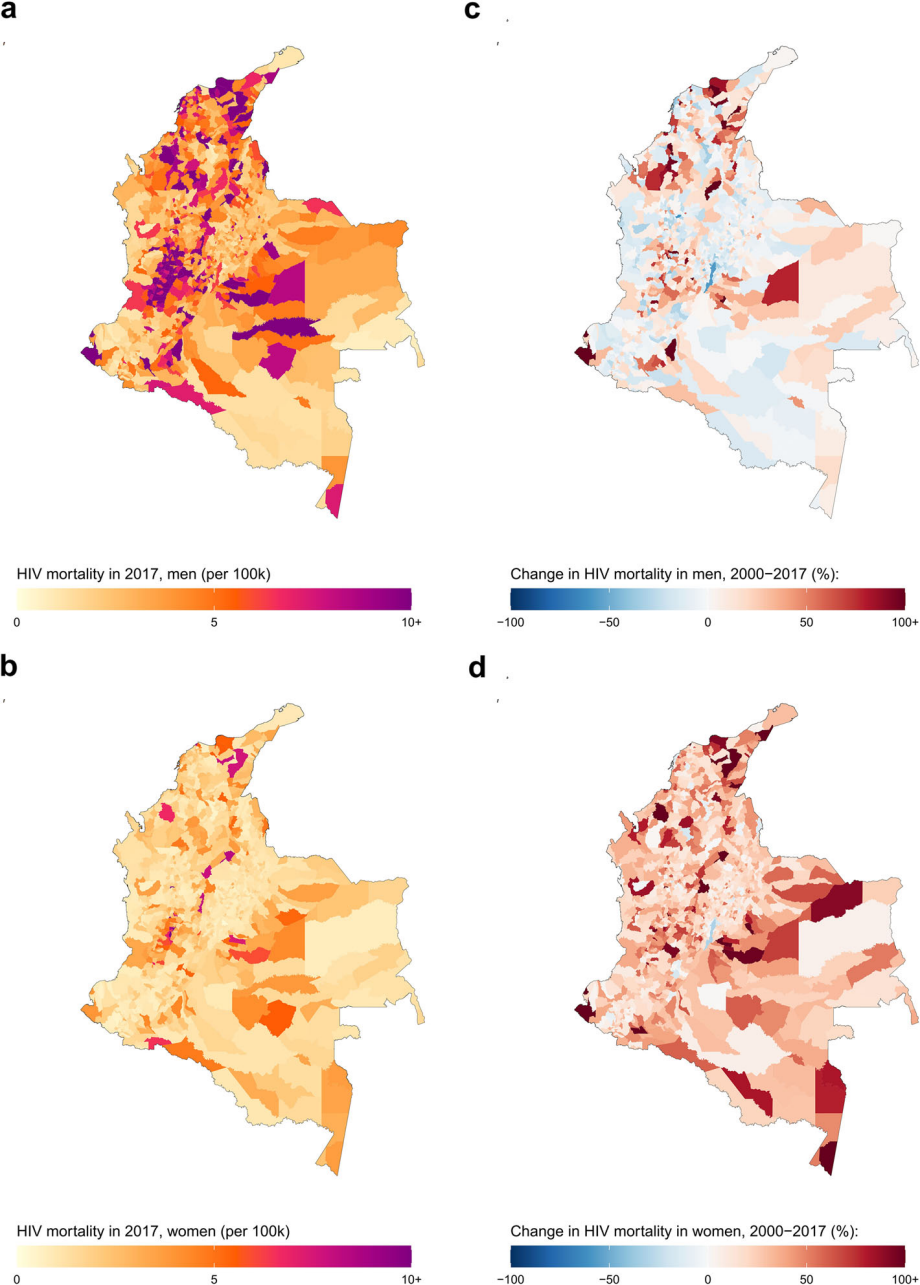
HIV mortality among men and women in Colombia by municipality, 2017. HIV mortality per 100,000 by municipality in Colombia in 2017 among men (**a**) and women (**b**). Relative change in HIV mortality between 2000 and 2017 among men (**c**) and women (**d**)

In 2017, Colombia’s estimated national HIV mortality rate for both sexes combined was 5.0 (4.8–5.3) deaths per 100,000 (7.8 [7.5–8.1] deaths for men, and 2.5 [2.3–2.7] deaths for women). Estimated male HIV mortality in 2017 varied over 75-fold: from 0.5 (0.2–0.9) deaths per 100,000 in the La Calera municipality, Cundinamarca department to 37.6 (26.4–50.9) deaths in the Chinchiná municipality, Caldas department (Fig. 2 and Additional file 1: Figure S11). Among women, estimated mortality varied over 86-fold, fluctuating from 0.2 (0.1–0.5) deaths per 100,000 in the La Calera municipality, Cundinamarca department to 17.2 (10.6–27.4) deaths in the La Virginia municipality, Risaralda department. Between 2000 and 2017, changes in national HIV mortality diverged by sex: estimated HIV mortality decreased among men by 19.8% (from 9.8 [9.3–10.2] deaths per 100,000 in 2000) but increased among women by 19.5% (from 2.1 [1.9–2.3] deaths per 100,000 in 2000). While national estimated HIV prevalence decreased among men and not women, HIV mortality increased in a majority of municipalities from 2000 to 2017 for both men and women. Estimated male HIV mortality increased in 594 (60.8%) municipalities, and five (1.6%) municipalities had a statistically significant increase in HIV mortality. Estimated female HIV mortality increased in 1081 (96.3%) municipalities and five (0.4%) municipalities had a statistically significant increase in HIV mortality. There was large variation in subnational changes in HIV mortality: estimated male HIV mortality ranged from a 361.7% increase in San Andrés de Tumaco municipality, Nariño department (from 2.8 [1.7–4.3] deaths per 100,000 in 2000 to 12.8 [9.4–17.1] deaths per 100,000 in 2017) to a 59.7% decrease in Barbosa municipality, Antioquia department (from 15.5 [9.4–24.5] deaths per 100,000 in 2000 to 6.2 [3.2–10.5] deaths per 100,000 in 2017). Among women, estimated relative change in HIV mortality at the municipality level varied from a 170% increase in Puerto Colombia municipality, Atlántico department (from 3 [1.5–5.4] deaths per 100,000 in 2000 to 8.2 [3.9–16.1] deaths per 100,000 in 2017) to a 31.2% decrease in Bogotá, D.C. municipality, Bogotá, D.C. department (from 1.6 [1.4–1.9] deaths per 100,000 in 2000 to 1.1 [0.9–1.3] deaths per 100,000 in 2017).

#### Costa Rica

**Fig. 3:**
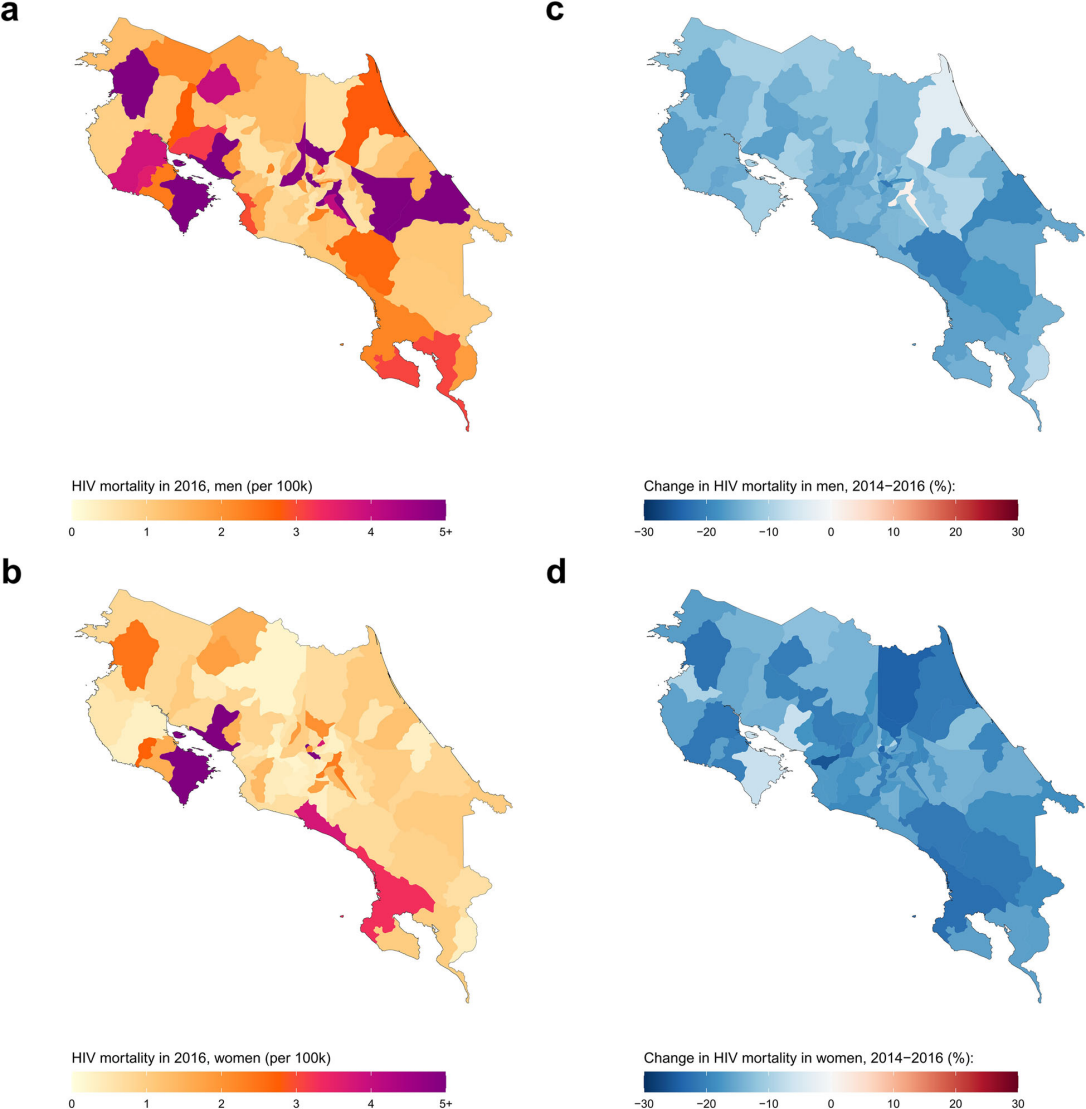
HIV mortality among men and women in Costa Rica by canton, 2016. HIV mortality per 100,000 by canton in Costa Rica in 2016 among men (**a**) and women (**b**). Relative change in HIV mortality between 2014 and 2016 among men (**c**) and women (**d**)

Among the six countries considered, Costa Rica had the lowest HIV mortality rates in the latest year of study, with an estimated national HIV mortality rate for both sexes combined in 2016 of 3.2 (2.5–3.9) deaths per 100,000 (4.9 [4.2–5.8] deaths for men, and 1.5 [1.1–2.3] deaths for women). At the canton level, we estimated male HIV mortality varied over 104-fold in 2016, from 0.5 (0.01–2.9) deaths per 100,000 in the Aserrí canton to 52.1 (42.1–63.2) deaths in the San José canton, San José province (Fig. 3 and Additional file 1: Figure S12). For women, mortality ranged over 40-fold, from 0.2 (0.0–1.5) deaths per 100,000 in the Atenas canton, Alajuela province, to 11.9 (7.8–17.4) deaths per 100,000 in the San José canton, San José province. Between 2014 and 2016, estimated national HIV mortality decreased by 18.5% among men (from 6.0 [5.1–7.1] deaths per 100,000 in 2014) and by 20% among women (from 1.9 [1.4–2.7] deaths per 100,000 in 2014). Estimated HIV mortality decreased in 80 (98.8%) municipalities for men and all 81 municipalities for women, though no canton for either sex registered a statistically significant decrease in HIV mortality. National temporal decreases were largely driven by the San José canton, San José province, which had the largest decrease in estimated male HIV mortality: 21.1%, from 65.9 [53.6–81.5] deaths per 100,000 in 2014 to 52.1 [42.1–63.2] deaths per 100,000 in 2016. From 2014 to 2016, estimated HIV mortality in San José canton, San José province, decreased among women by 22.7% (from 15.3 [10.4–22.9] deaths per 100,000 in 2014 to 11.9 [7.8–17.4] deaths per 100,000 in 2016).

#### Ecuador

**Fig. 4:**
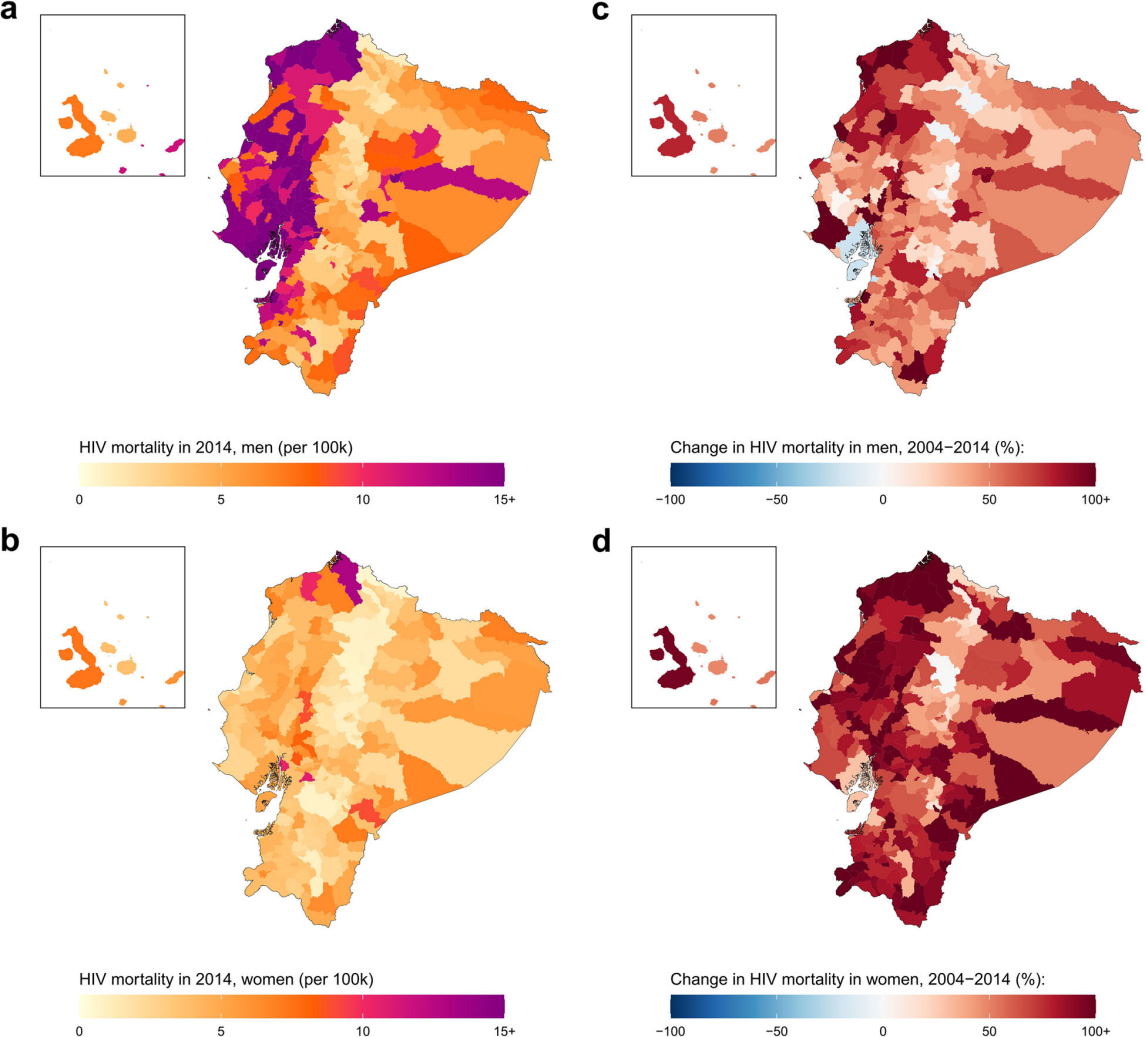
HIV mortality among men and women in Ecuador by canton, 2014. HIV mortality per 100,000 by canton in Ecuador in 2014 among men (**a**) and women (**b**). Relative change in HIV mortality between 2004 and 2014 among men (**c**) and women (**d**)

In 2014, Ecuador had the highest estimated national HIV mortality rate for both sexes combined among the six countries investigated: 7.0 (6.5–7.6) deaths per 100,000 (10.9 [10.1–11.7] deaths for men, and 3.4 [3.0–3.8] deaths for women). Estimated male HIV mortality varied among cantons from 1.1 (0.4–2.4) deaths per 100,000 in the Tulcán canton, Carchi province, to 50.7 (29.0–81.6) deaths per 100,000 in the Palestina canton (Fig. 4 and Additional file 1: Figure S16). For women, estimated HIV mortality ranged from 0.6 (0.2–1.3) deaths per 100,000 in the Tulcán canton, Carchi province, to 13.0 (4.3–29.8) deaths per 100,000 in the San Lorenzo canton, Esmeraldas province. Between 2004 and 2014, estimated national HIV mortality increased by 28.3% among men (from 8.5 [7.8–9.2] deaths per 100,000 in 2004) and increased by 63.2% among women (from 2.1 [1.8–2.4] deaths per 100,000 in 2004). Estimated male HIV mortality increased in 216 (96.4%) cantons, and three [1.3%] municipalities had a significant increase in HIV mortality. Estimated female HIV mortality increased in 223 (99.6%) municipalities and one [0.4%] canton had a statistically significant increase in HIV mortality. Among men, estimated change in HIV mortality at the canton level ranged from a 239.7% increase in Río Verde canton, Esmeraldas province (from 9.1 [2.9–24.4] deaths per 100,000 in 2004 to 31 [9.5–79.1] deaths per 100,000 in 2014), to a 42.7% decrease in Huaquillas canton, El Oro province (from 8.3 [3.9–15.9] deaths per 100,000 in 2004 to 4.7 [1.8–10] deaths per 100,000 in 2014). Among women, estimated relative change in HIV mortality at the canton level varied from a 199.9% increase in Río Verde canton, Esmeraldas province (from 3.4 [0.9–10.2] deaths per 100,000 in 2004 to 10.2 [1.7–34.6] deaths per 100,000 in 2014), to essentially no change in Latacunga canton, Cotopaxi province (0.6 [0.3–1.2] deaths per 100,000 in 2004 compared to 0.6 [0.3–1.3] deaths per 100,000 in 2014).

#### Guatemala

**Fig. 5:**
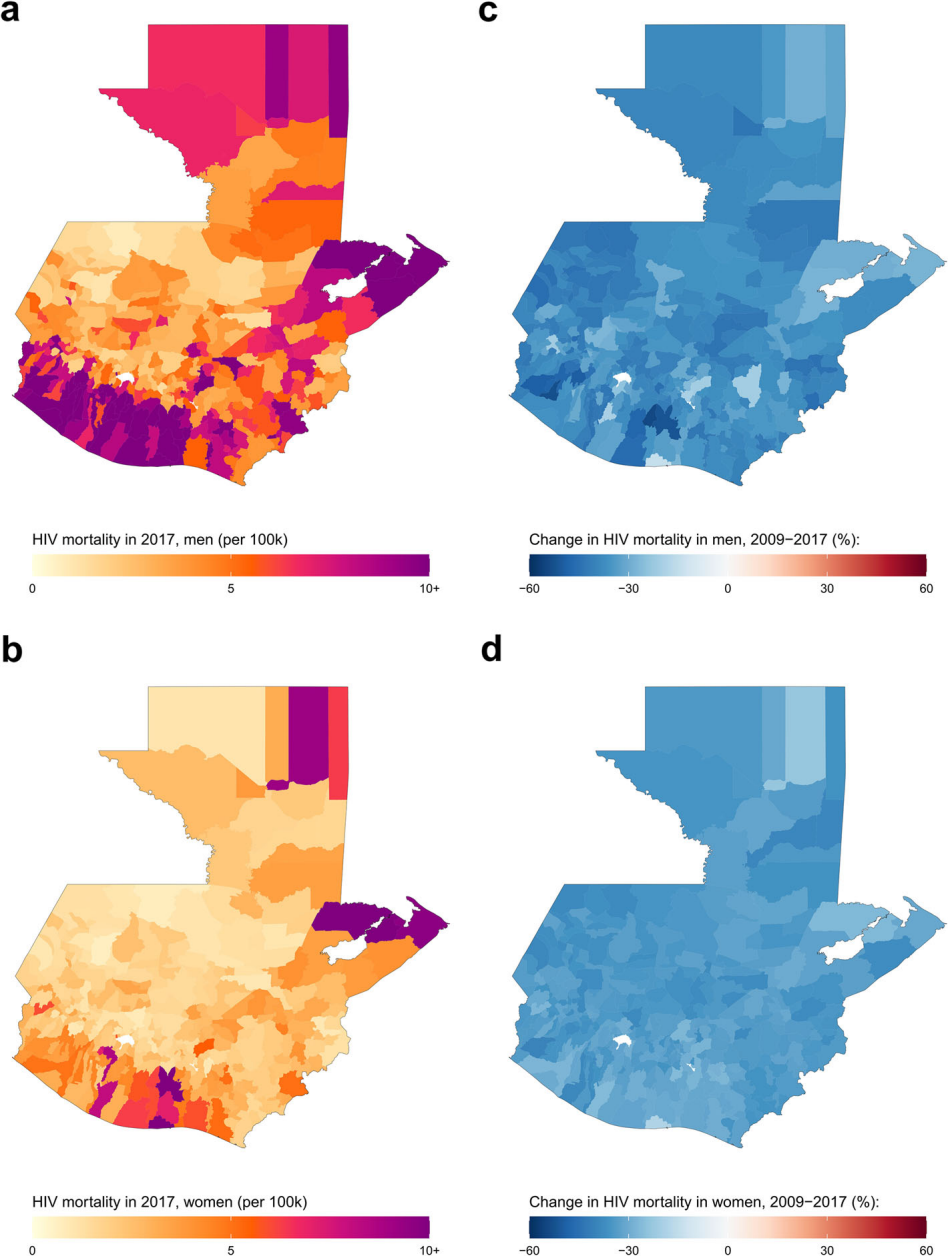
HIV mortality among men and women in Guatemala by municipality, 2017. HIV mortality per 100,000 by municipality in Guatemala in 2017 among men (**a**) and women (**b**). Relative change in HIV mortality between 2009 and 2017 among men (**c**) and women (**d**)

In Guatemala, estimated national HIV mortality for both sexes combined in 2017 was 4.6 (4.1–5.1) deaths per 100,000 (6.8 [6.2–7.4] deaths for men and 2.8 [2.4–3.1] deaths for women). At the municipality level, estimated HIV mortality for men varied from 0.8 (0.2–2.1) deaths per 100,000 in the Santa Cruz Barillas municipality, Huehuetenango department to 38.6 (24.5–57.5) deaths per 100,000 in the San José municipality, Escuintla department (Fig. 5 and Additional file 1: Figure S11). For women, estimated HIV mortality ranged from 0.7 (0.2–1.5) deaths per 100,000 in the Chiantla municipality, Huehuetenango department to 20.3 (12.3–31.8) deaths per 100,000 in the San José municipality, Escuintla department. Between 2009 and 2017, estimated national HIV mortality decreased by 36.9% among men (from 10.8 [9.9–11.7] deaths per 100,000 in 2009) and by 33.5% among women (from 4.1 [3.7–4.6] deaths per 100,000 in 2009). Unlike in the other countries considered, estimated HIV mortality decreased in all 340 municipalities for both men and women though only two [0.6%] municipalities for men and no municipalities for women had a statistically significant decrease in HIV mortality. Among men, estimated relative change in HIV mortality at the municipality level ranged from a 14.4% decrease in San José municipality, Escuintla department (from 45 [30.2–65.5] deaths per 100,000 in 2009 to 38.6 [24.5–57.5] deaths per 100,000 in 2017) to a 54% decrease in Siquinalá municipality, Escuintla department (from 25.4 [14.3–41.3] deaths per 100,000 in 2009 to 11.7 [6.5–19.7] deaths per 100,000 in 2017). Estimated change in female HIV mortality at the municipality level varied from an 18.5% decrease in San José municipality, Escuintla department (from 24.9 [15.9–38.6] deaths per 100,000 in 2009 to 20.3 [12.3–31.8] deaths per 100,000 in 2017) to a 39.9% decrease in Coatepeque municipality, Quetzaltenango department (from 6.9 [4.4–10.3] deaths per 100,000 in 2009 to 4.1 [2.6–6.6] deaths per 100,000 in 2017).

#### Mexico

**Fig. 6:**
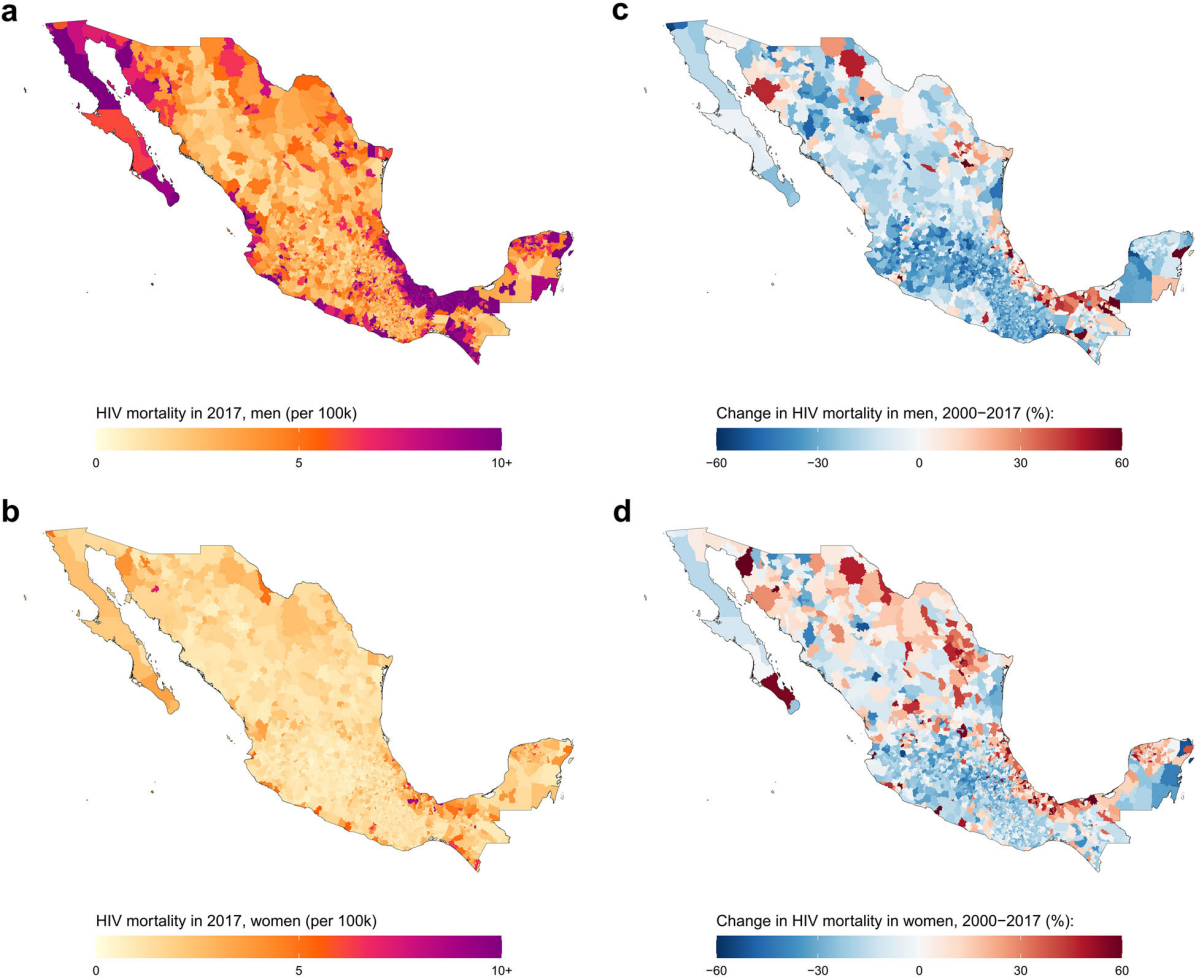
HIV mortality among men and women in Mexico by municipality, 2017. HIV mortality per 100,000 by municipality in Mexico in 2017 among men (**a**) and women (**b**). Relative change in HIV mortality between 2000 and 2017 among men (**c**) and women (**d**)

In Mexico, the estimated national HIV mortality rate for both sexes combined in 2017 was 4.3 (4.2–4.4) deaths per 100,000 (6.9 [6.7–7.1] deaths per 100,000 for men, and 1.9 [1.8–2.0] for women). At the municipality level, estimated HIV mortality for men in 2017 varied over 53-fold: from 0.8 (0.4–1.3) deaths per 100,000 in the San Salvador Atenco municipality, Mexico state to 42.6 (29.1–61.4) deaths in the Tlacotalpan municipality, Veracruz state (Fig. 6 and Additional file 1: Figure S12). For women, estimated HIV mortality ranged over 47-fold from 0.3 (0.2–0.6) deaths per 100,000 in the Texcoco municipality, Mexico state to 14.2 (8.8–22.0) deaths per 100,000 in the San Juan Cancuc municipality, Chiapas state. Between 2000 and 2017, estimated national HIV mortality decreased by 23.5% among men (from 9.0 [8.8–9.3] deaths per 100,000 in 2000) and by 5.2% among women (from 2.0 [1.9–2.1] deaths per 100,000 in 2000). Estimated male HIV mortality decreased in 2048 (83.3%) municipalities, and 32 [1.3%] municipalities had a statistically significant decrease in HIV mortality. Estimated female HIV mortality decreased in 1683 (68.5%) municipalities and 4 [0.2%] municipalities had a significant decrease in HIV mortality. Among men, estimated change in HIV mortality from 2000 to 2017 ranged from a 162.4% increase in the Solidaridad and Tulum municipalities, Quintana Roo state (from 6.8 [4.9–9.3] deaths per 100,000 in 2000 to 17.9, [14.5–21.6] deaths per 100,000 in 2017) to a 61.9% decrease in Playas de Rosarito municipality, Baja California state (from 19.3 [13.6–27.2] deaths per 100,000 in 2000 to 7.4 [5.1–10.2] deaths per 100,000 in 2017). For women, estimated relative change in HIV mortality at the municipality level varied from a 110.3% increase in Coatzacoalcos municipality, Veracruz state (from 4.5 [3.5–5.8] deaths per 100,000 in 2000 to 9.5 [7.5–11.8] deaths per 100,000 in 2017) to a 54.9% decrease in Zapopan municipality, Jalisco state (from 2.5 [1.9–3.3] deaths per 100,000 in 2000 to 1.1 [0.8–1.5] deaths per 100,000 in 2017).

### Concentrated deaths due to HIV

**Fig. 7:**
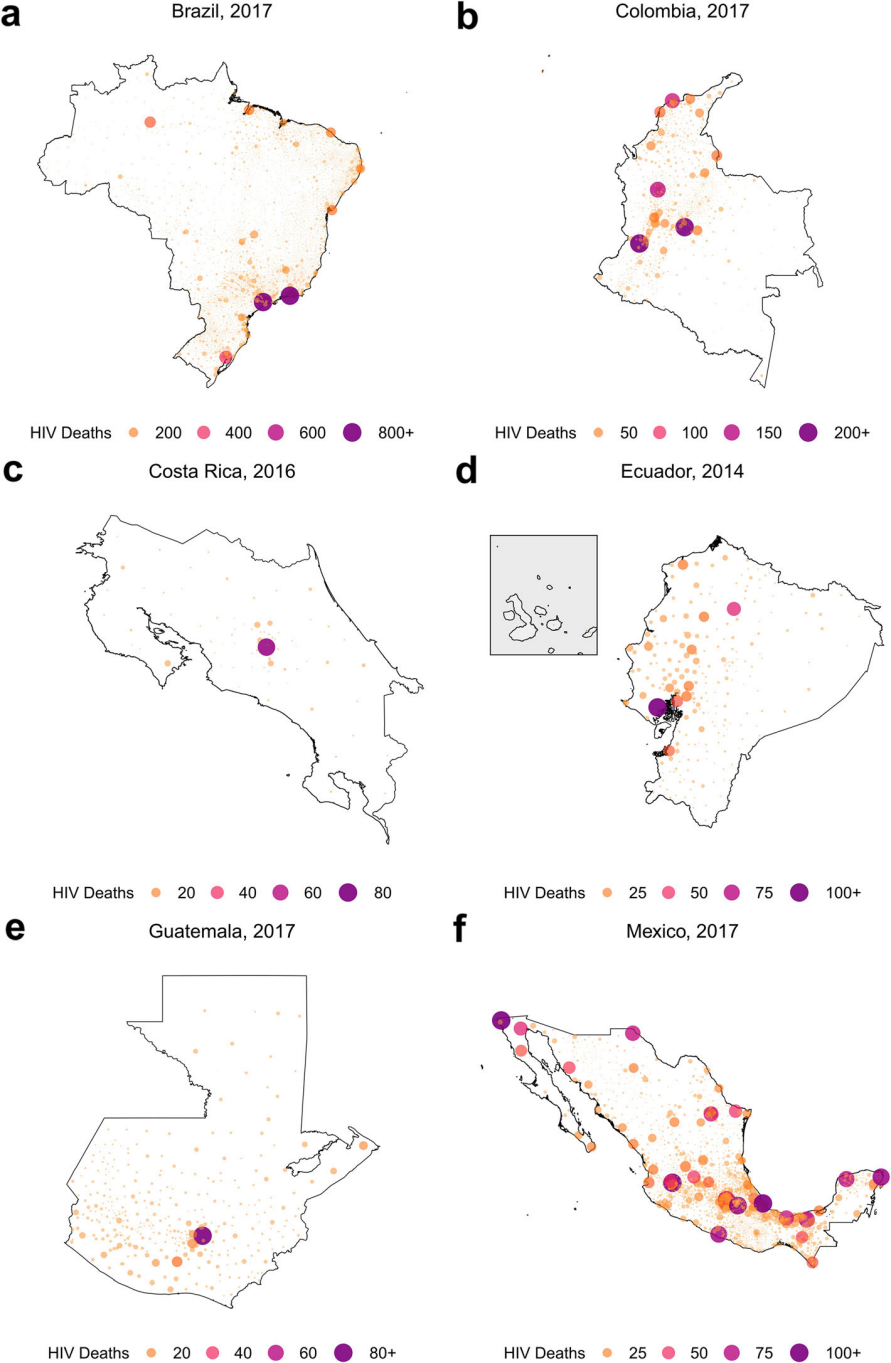
Number of HIV deaths in latest year of study, by municipality. Estimated number of HIV deaths by municipality in the latest year of study: 2017 in Brazil (**a**), 2017 in Colombia (**b**), 2016 in Costa Rica (**c**), 2014 in Ecuador (**d**), 2017 in Guatemala (**e**), 2017 in Mexico (**f**). Color and size are proportional to estimated HIV deaths

We estimated that a large proportion of HIV deaths were concentrated in a small number of geographical areas with large populations. In all countries, over half of the HIV deaths were located in less than 10% of municipalities in the latest year of study (Fig. 7). In Colombia, over half of all HIV deaths in 2017 were located in just 1.2% (14 of 1122) of municipalities that contain 37.0% of the total population, and in Guatemala in 2017, over half the HIV deaths were spread out over 9.4% (32 of 340) of municipalities that contain 33.4% of the population. Several countries contained single municipalities that contributed a large proportion of national HIV deaths: for example, in Costa Rica in 2016, 45.9% of all HIV deaths were located in San José canton, San José province, compared to 4.6% of the total population. Mexico had a greater spread of HIV deaths across municipalities; in 2017 the area with the highest proportion of HIV deaths was Tijuana municipality, Baja California state, which amounted to 3.2% of the total deaths compared to 1.5% of the population. There was higher HIV mortality among men as compared to women in all countries, ranging from 64.7% of all deaths concentrated among men in Brazil in 2017, to 77.6% of all deaths concentrated among men in Mexico in 2017.

### Absolute and relative inequality over time

**Fig. 8:**
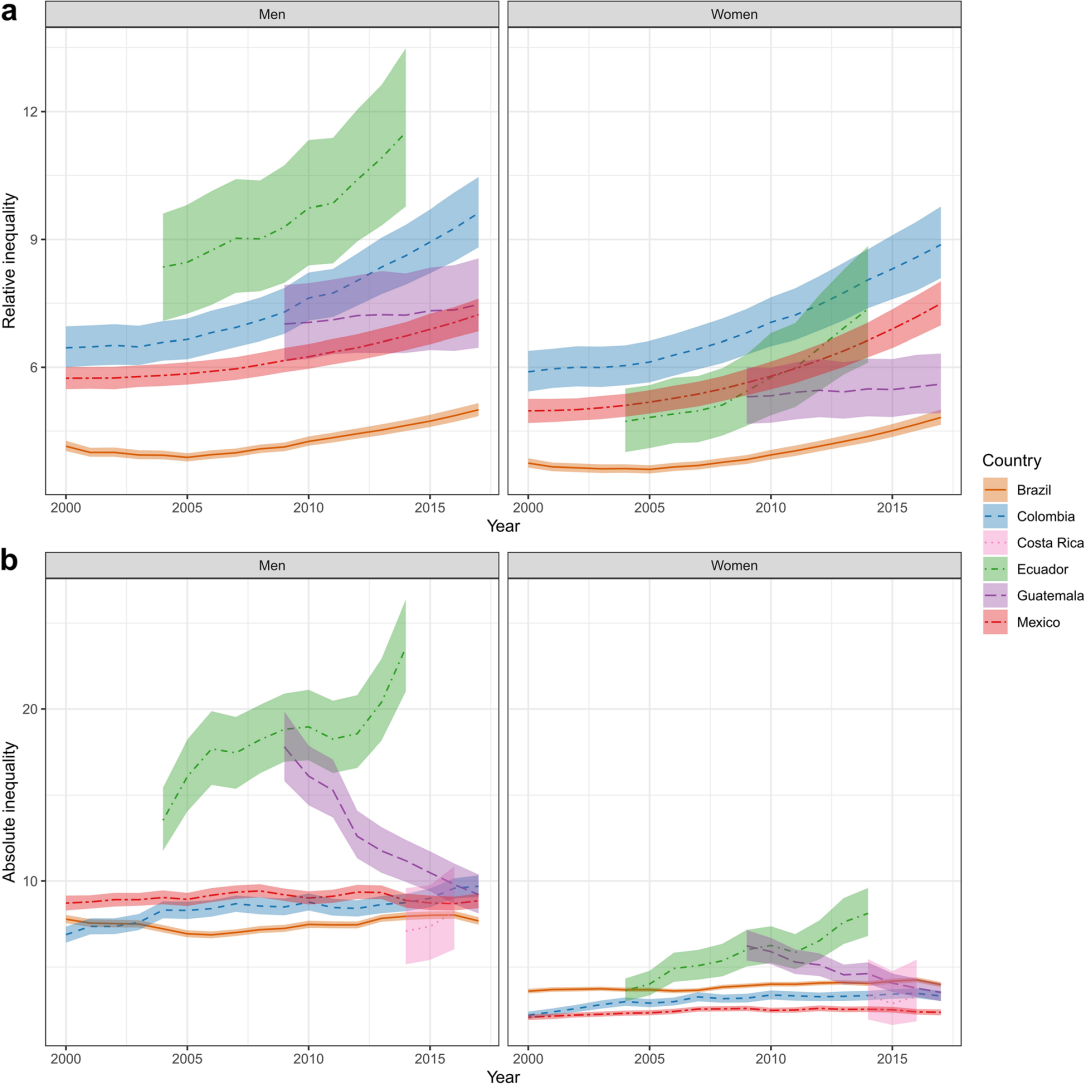
Relative and absolute inequality among municipalities in HIV mortality. **a** Relative inequality, defined as the ratio of estimated HIV mortality for municipalities in the 90th percentile versus 10th percentile, by year with 95% uncertainty intervals. Costa Rica is omitted from this panel because its estimated relative inequality was > 50. **b** Absolute inequality, defined as the difference in HIV mortality rates for municipalities in the 90th versus 10th percentile, by year with 95% uncertainty intervals. Selected countries are differentiated by color and line type

Relative inequality—the mortality rate ratio for municipalities in the 90th percentile versus those in the 10th percentile—varied from 5.0 (4.9–5.2) and 4.8 (4.6–5.0) among men and women in Brazil in 2017, to 63.4 (31.6–113.0) and 167.6 (54.6–403.5) among men and women in Costa Rica in 2016 (Fig. 8). The estimated relative geographic inequality in HIV mortality increased in all countries from the first to the last year of study, and this increase was statistically significant in all countries barring Guatemala and Costa Rica. The largest percent increase in relative inequality for each sex over the study period was 49.0% in Colombian men (from 6.5 [6.0–7.0] in 2000 to 9.6 [8.8–10.5] in 2017) and 55.9% in Ecuadorian women (from 4.7 [4.0–5.5] in 2004 to 7.4 [6.1–8.8] in 2014).

Absolute inequality—the difference between the mortality rate in the 90th percentile and the 10th percentile—showed less temporal variation in Brazil, Mexico, and Costa Rica: the difference in estimated absolute inequality between the first and last year of study was less than 1.5 deaths per 100,000 among both men and women. Male absolute inequality increased in Colombia by 40.9% (from 6.9 [6.4–7.4] in 2000 to 9.7 [9.1–10.4] in 2017) and in Ecuador by 74.1% (from 13.5 [11.8–15.4] in 2004 to 23.6 [21.0–26.4] in 2014), while male absolute inequality decreased in Guatemala by 48.2% (from 17.8 [15.8–19.8] in 2009 to 9.2 [8.1–10.4] in 2017). Female absolute inequality increased in Colombia by 50.8% (from 2.2 [2.0–2.4] in 2000 to 3.3 [3.0–3.6] in 2017) and Ecuador by 123.9% (from 3.6 [3.0–4.3] in 2004 to 8.1 [6.8–9.6] in 2014), while female absolute inequality decreased in Guatemala by 43.5% (from 6.2 [5.4–7.2] in 2009 to 3.5 [3.0–4.1] in 2017). For both men and women, increases in absolute relative inequality in Colombia and Ecuador, as well as decreases in absolute inequality in Guatemala, were statistically significant.

### Local disparities in median age group among those who died from HIV

**Fig. 9:**
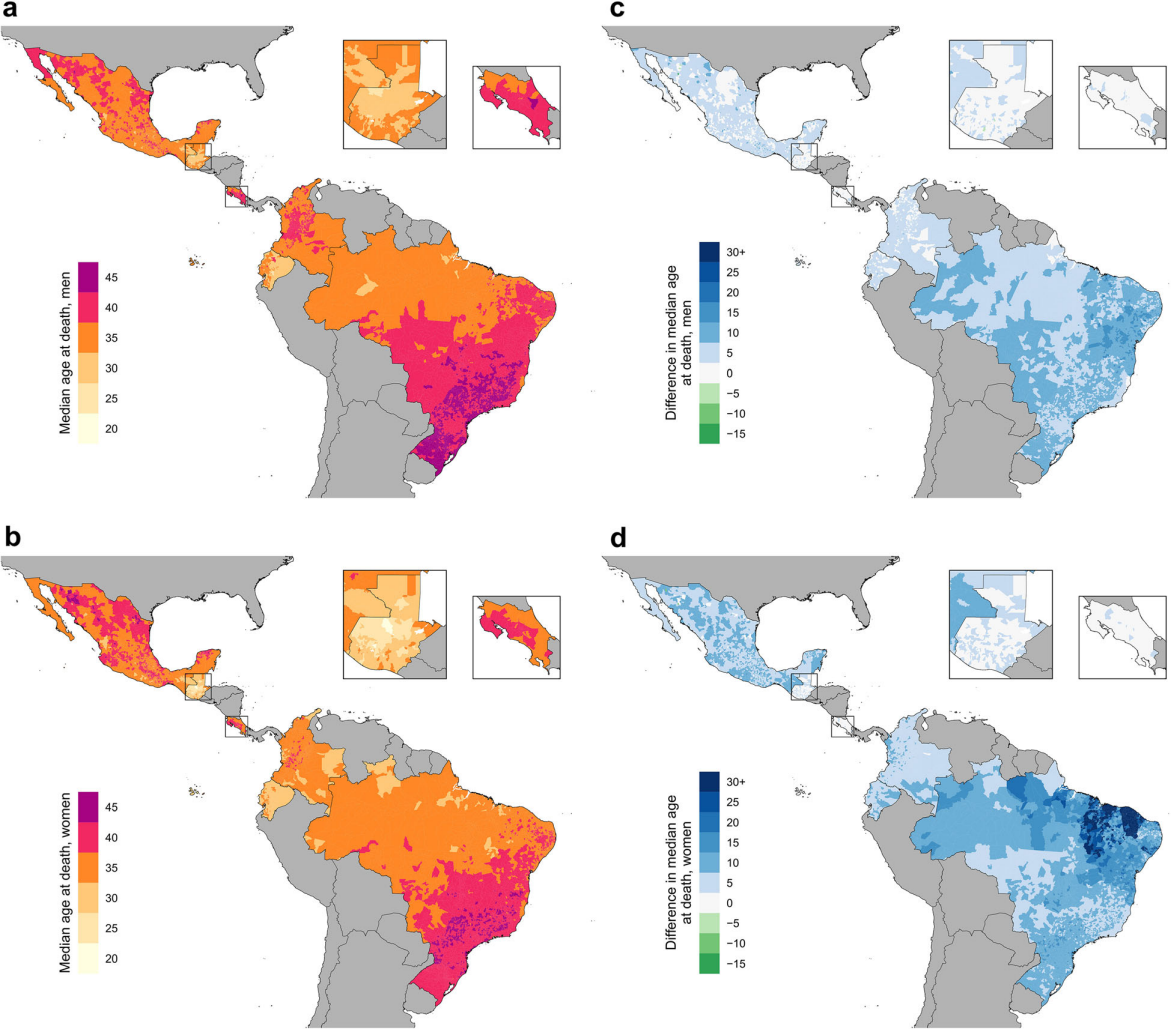
Estimated median age group among those who died from HIV, by municipality. Estimated median age of death among men (**a**) and women (**b**) who died from HIV in the last year of study in selected countries: 2017 in Brazil, Colombia, Guatemala, and Mexico, 2016 in Costa Rica, and 2014 in Ecuador. Estimated difference in median age of death among men (**c**) and women (**d**) who died from HIV from first year to last year of study in selected countries (2000–2017 in Brazil, Colombia, and Mexico, 2009–2017 in Guatemala, 2014–2016 in Costa Rica, and 2004–2014 in Ecuador)

The estimated median age group among men who died varied substantially at the municipality level in the latest year of study: by 15 years in Brazil, Ecuador, and Mexico, and by 10 years in Colombia, Costa Rica, and Guatemala (Fig. 9). Among women, estimated median age group among those who died in the latest year of study varied at the municipality level by 15 years in Brazil, Guatemala, and Mexico, by 10 years in Colombia, and by only 5 years in Costa Rica and Ecuador. Differences in median age group among those who died also shifted over time. In Brazil, the estimated median age group among male decedents rose in 99.6% of municipalities from 2000 to 2017, while in Guatemala only 21.5% of municipalities saw an increase in estimated median age group among male decedents from 2009 to 2017. An increase in estimated median age was also observed among women: in Mexico, Ecuador, Colombia, and Brazil, the median age group among female decedents rose in > 97% of all municipalities in each country. Additional file 1: Figure S16-S21 show estimated HIV mortality by age group and sex for each country in the last year of study.

## Discussion

There are few past analyses that assess HIV mortality in Latin America using VR data at a subnational level, largely due to the statistical challenges of incorporating incomplete VR systems and estimating mortality in areas with small populations and small numbers of deaths. The few analyses that integrate estimates of VR completeness into their modeling framework are often done at the national or state level or are limited to a single country and year [15–18]. In this analysis, we expand on previously described methods [31] that include prior estimates of VR completeness and demonstrate the utility of estimates that combine uncertainty from both incomplete registration systems and from statistical methods designed to leverage information across space, time, and age to inform mortality rates in areas with small numbers of HIV deaths.

Our estimates revealed large-scale spatial heterogeneity in HIV mortality across the six Latin American countries considered in our analysis. We also reveal divergent national trends in HIV mortality in the six countries across the study period, and variable relative change within countries at the municipality level. From the first to the last year of study, HIV mortality decreased among men and women in all countries, with the exception of women in Colombia from 2000 to 2017 and both men and women in Ecuador from 2004 to 2014. Despite the progress in reducing HIV mortality among both sexes at the national level in Brazil, Guatemala, Costa Rica, and Mexico, inequalities in municipality-level HIV mortality persist and relative inequality increased over time in all countries. This analysis highlights uneven progress towards reducing HIV mortality and reaching UNAIDS Fast-Track goals, and emphasizes an alarming trend in Ecuador, where over 95% of cantons experienced increases in estimated mortality among both sexes from 2004 to 2014. Nonetheless, it also underlines stories of success: all countries contained municipalities with an estimated decrease in HIV mortality. Further evaluating municipalities with the greatest decreases in HIV mortality within a country may help decision-makers recognize successful strategies that could be implemented in municipalities experiencing increases or slower declines.

There are likely a multitude of factors that contribute to the spatial and temporal patterns in HIV mortality observed in our analysis. Consistent with past analyses, we found higher rates of HIV mortality among men compared to women and slightly different spatial patterns by sex [15, 48, 49]. The consistently elevated levels of mortality among men is likely partially because men who have sex with men (MSM) continue to be one of the populations with the highest prevalence throughout Latin America, and a group that suffers a higher level of stigma and discrimination [48, 50–52]. These trends may also reflect prevalent gender norms [49], unequal access to timely diagnosis and treatment [48], or differences in disease burden from comorbidities between genders [1]. The spatial distribution of high-risk groups possibly also contributes to the HIV epidemic remaining concentrated in large urban centres [6]. For example, one important driver of spatial differences in HIV mortality is prison populations, where HIV transmission is high due to overcrowding, violence, and lack of information on the risk of HIV acquisition [53]. In Brazil, there is evidence of low adherence to antiretroviral therapy (ART) and a higher proportion of primary and secondary resistance among prison populations, which are predominantly male [54]. Municipalities with large prison populations—such as several in the state of Sao Paulo, Brazil—show higher levels of HIV mortality rates and number of deaths due to HIV. Another potential driver of spatial heterogeneity is population migration. Political conflicts and economic hardships across the region, notably in Venezuela and Central America, have fostered waves of migration that can affect HIV prevention, treatment, and care program [55]. Furthermore, difficulties in acquiring HIV treatment and ART shortages spurs regional migration that can differentially impact HIV care and control programs in bordering countries [55, 56].

A key driver of temporal trends in HIV mortality is the implementation of ART treatment programs, which have been incorporated to varying degrees in all Latin American countries and generally led to increases in ART coverage [55]. ART treatment is often a central focus of national HIV programs, but differences in priority and ability to commit resources have likely impacted progress in reducing HIV mortality. Access to HIV treatment for people living with HIV is country-dependent and has shifted over time. Brazil was the first middle-income country to offer free ART treatment to people living with HIV (PLHIV) in 1996 [57], with Costa Rica following soon after in 1998. Within the ensuing decade, Mexico [58] and Colombia [59] adopted similar policies of universal treatment, which may have contributed to the observed reduction in national HIV mortality as these programs matured. In recent years, Guatemala has also expanded ART treatment options through joint support from the national government and The Global Fund to Fight AIDS, Tuberculosis, and Malaria [60]. Given the concentration of HIV in urban high-risk groups, many national programs—such as those in Costa Rica and Guatemala—have emphasized a combination prevention strategy that focus on HIV testing, STI diagnosis, and linkage to care in vulnerable populations [61]. In Ecuador, the only country in our analysis where we estimated increases in national HIV mortality among men and women over the study period, there has historically been a paucity of information available on research findings concerning HIV/AIDS burden [62]. Notably, our analysis in Ecuador only extends to 2014 and patterns of HIV mortality may have changed with the recent emphasis on community testing and treatment in metropolitan areas [63]. While all countries have expanded access to treatment and documented increases in ART coverage, albeit at different time periods, persistent disparities in access to quality health services and adherence to ART remain [6, 64, 65] and may contribute to differences in HIV mortality declines observed in this analysis. Further, in all countries in our analysis, communities with socioeconomic and health disadvantages—such as indigenous communities, sex workers, and transgender populations—often have unequal access to treatment and are an emerging or establish public health priority [66, 67].

Our analysis also revealed substantial variation in median age group among those who died from HIV at the municipality level, and an increase over time in median age group among those who died of HIV. These results agree with past research that demonstrates an accelerating growth in the number of people living with HIV that are above 50 years of age [68, 69]. Several countries, notably Brazil and Mexico, contained municipalities with a 15-year difference in median age groups among men and women who died from HIV in the latest year of study. While attributing these trends to specific drivers is outside the scope of this analysis, there are several factors that could influence an increase in median age of death, including changes in HIV incidence in specific age groups [70], increases in life expectancy among people living with HIV [70], access to ART [71], migration, and the age distribution of the population across the study period.

This analysis provides novel subnational estimates of HIV mortality that convey important information to policymakers and could inform future action. Knowledge of local differences in HIV mortality can help guide scale-up of ART where mortality might reflect suboptimal coverage. Our estimates highlight how deaths due to HIV are concentrated in a low proportion of municipalities. In the longer term, HIV mortality measures could be used to highlight areas that might benefit from programmatic interventions that target HIV prevention such as pre-exposure prophylaxis (PrEP). As countries in Latin America consider expanding access to PrEP, studies have demonstrated that prioritization of PrEP to those at highest risk could save money and lives [72, 73]. Cost-effective interventions are especially important in Guatemala, Ecuador, Colombia, and Costa Rica, where HIV programs depend on donor funding [74]. Furthermore, subnational differences in HIV burden have already been used to develop localized strategies for HIV prevention and elimination in sub-Saharan Africa [75, 76].

### Limitations

This analysis is subject to a number of limitations. First, the VR data that inform our estimates are subject to misclassification biases. HIV is generally under-reported as a cause of death [23]. While the HIV correction methodology from the GBD employed in this analysis corrects for biases in HIV deaths classified to other underlying causes of death, there may be additional country-specific biases not addressed by our methodological approach. While we matched decedents to their municipality of residence as provided by the VR mortality databases, these data could overrepresent urban areas with larger health care facilities where individuals may have died after seeking treatment. It is possible that in some cases these individuals may have been mistakenly recorded as residing in that municipality if their municipality of residence was not available. Second, our method for correcting for incomplete VR across space and time makes several crucial assumptions. Our analysis incorporates estimates of VR completeness for children under 15 and adults 15 and over based on previous analyses, but VR completeness may vary within these age ranges. Further, we use the geographic variation in completeness identified by comparing reported under-5 all-cause deaths to previous estimates, and the variation in under-5 mortality may not be comparable to patterns in adult VR incompleteness. Additionally, variation in all-cause completeness may differ from patterns in HIV-specific VR completeness. Third, we use population estimates from WorldPop in this analysis that are subject to error, especially in sparsely populated areas. While WorldPop estimates include census data as inputs [77], depending on timing and data accessibility, estimates may differ from the underlying census measures and may not utilize the most recent census or the most detailed tabulations. Fourth, population migration in response to political conflict or economic instability in the region, including Central America and Venezuela, may not be properly captured in WorldPop estimates or recorded in vital registration systems. Fifth, our analysis is subject to large uncertainty. This reflects uncertainty both due to the small number of HIV deaths at the municipality level and the need to estimate completeness. While we believe this method better captures major sources of uncertainty, care must be taken when interpreting results. Sixth, we use custom shapefiles that are matched to country-level administrative subdivisions, and differences in administrative divisions between GAUL [35] or the Humanitarian Data Exchange [36] and an individual country’s designation of administrative areas may affect the accuracy of results, especially in our estimates of the number of HIV deaths by municipality. Seventh, our small area estimation models smooth over space and time by making assumptions about the temporal and spatial structure of HIV mortality that may not always hold. Eighth, VR data availability varied across the countries selected in our analysis, and comparison between temporal trends in HIV mortality may be difficult to assess for countries with different years of data availability. Finally, it is difficult to directly assess violations of our modeling assumptions or quality issues in the underlying data sources given that VR completeness cannot be verified. Nonetheless, comparisons to GBD national estimates (Additional file 1: Figure S4-S9) provide reassurance in overall country trends.

### Future directions

There is considerable opportunity to expand this analysis. ﻿First, access to VR data over more years of study, or in neighboring countries in Latin America, could provide valuable benchmarks for more direct comparisons and allow additional information across space and time to potentially improve our models. Our current study uses four available covariates that serve as proxies for urbanization and development, but in the future, availability of other drivers of HIV mortality at the municipality level such as socioeconomic status, healthcare infrastructure, high-risk group concentration, and ART treatment availability could improve our estimates. Further, the technique we used to include uncertainty and information on subnational VR completeness could be extended to other countries where VR systems are not complete. Finally, this small area estimation framework could be used to estimate all-cause and cause-specific mortality due to other causes at local levels in the six modeled Latin American countries.

## Conclusion

Our analysis finds large-scale variation in HIV mortality among municipalities in six Latin American countries, both in the latest year of study as well as over the entire study period. Our estimates demonstrate the need to assess HIV burden at a granular geographic scale in Latin America, given that the HIV epidemic is concentrated in high-risk groups and select urban areas. The methods developed in this analysis provide a framework for incorporating prior information on VR completeness into subnational estimates of HIV burden. This analysis could be used to identify areas that have successfully reduced HIV mortality and areas of high HIV burden, as well as to inform the rollout of preventive interventions that are required to help countries progress towards achieving UNAIDS targets and advance health equity.

## Supplementary Information

**Additional file 1:.** Supplemental methods, GATHER checklist, Supplemental Figure S1-S21, and Supplemental Tables S1-S4. Figure S1. Analytical process overview. Figure S2. Analytical process for VR data. Figure S3. Analytical process overview for VR completeness priors. Figure S4. Model alignment with GBD, Brazil. Figure S5. Model alignment with GBD, Colombia. Figure S6. Model alignment with GBD, Costa Rica. Figure S7. Model alignment with GBD, Ecuador. Figure S8. Model alignment with GBD, Guatemala. Figure S9. Model alignment with GBD, Mexico. Figure S10. Mean and uncertainty in estimated HIV mortality in Brazil, 2017. Figure S11. Mean and uncertainty in estimated HIV mortality in Colombia, 2017. Figure S12. Mean and uncertainty in estimated HIV mortality in Costa Rica, 2016. Figure S13. Mean and uncertainty in estimated HIV mortality in Ecuador, 2014. Figure S14. Mean and uncertainty in estimated HIV mortality in Guatemala, 2017. Figure S15. Mean and uncertainty in estimated HIV mortality in Mexico, 2017. Figure S16. Estimated HIV mortality in Brazil by age group, 2017. Figure S17. Estimated HIV mortality in Colombia by age group, 2017. Figure S18. Estimated HIV mortality in Costa Rica by age group, 2016. Figure S19. Estimated HIV mortality in Ecuador by age group, 2014. Figure S20. Estimated HIV mortality in Guatemala by age group, 2017. Figure S21. Estimated HIV mortality in Mexico by age group, 2017. Table S1: Merged municipalities by country to form stable geographical units. Table S2: Vital Registration data. Table S3: Covariate data sources. Table S4: National HIV mortality rates among men and womenfwil.

## Data Availability

Our study follows the Guidelines for Accurate and Transparent Health Estimates Reporting (GATHER). Estimates can be further explored at national, state, and municipality level by age group, sex, and year through our online visualization tools (https://vizhub.healthdata.org/lbd/hiv-mort-la). The source code used to generate estimates, as well as the outputs of the study (including full sets of estimates at the state and municipality levels), are publicly available online via the Global Health Data Exchange (http://ghdx.healthdata.org/record/ihme-data/latin-america-hiv-mortality-estimates-2000-2017). All maps presented in this study were generated by the authors and no permissions are required to publish them.
