# Supplementary material for: Mapping subnational HIV mortality in six Latin American countries with incomplete vital registration systems
Source: BMC Med. 2021 Jan 8;19:4. doi: 10.1186/s12916-020-01876-4 (PMC7791645; doi:10.1186/s12916-020-01876-4)
Supplement: Supplementary file 1 — Additional file 1:. Supplemental methods, GATHER checklist, Supplemental Figure S1-S21, and Supplemental Tables S1-S4. Figure S1. Analytical process overview. Figure S2. Analytical process for VR data. Figure S3. Analytical process overview for VR completeness priors. Figure S4. Model alignment with GBD, Brazil. Figure S5. Model alignment with GBD, Colombia. Figure S6. Model alignment with GBD, Costa Rica. Figure S7. Model alignment with GBD, Ecuador. Figure S8. Model alignment with GBD, Guatemala. Figure S9. Model alignment with GBD, Mexico. Figure S10. Mean and uncertainty in estimated HIV mortality in Brazil, 2017. Figure S11. Mean and uncertainty in estimated HIV mortality in Colombia, 2017. Figure S12. Mean and uncertainty in estimated HIV mortality in Costa Rica, 2016. Figure S13. Mean and uncertainty in estimated HIV mortality in Ecuador, 2014. Figure S14. Mean and uncertainty in estimated HIV mortality in Guatemala, 2017. Figure S15. Mean and uncertainty in estimated HIV mortality in Mexico, 2017. Figure S16. Estimated HIV mortality in Brazil by age group, 2017. Figure S17. Estimated HIV mortality in Colombia by age group, 2017. Figure S18. Estimated HIV mortality in Costa Rica by age group, 2016. Figure S19. Estimated HIV mortality in Ecuador by age group, 2014. Figure S20. Estimated HIV mortality in Guatemala by age group, 2017. Figure S21. Estimated HIV mortality in Mexico by age group, 2017. Table S1: Merged municipalities by country to form stable geographical units. Table S2: Vital Registration data. Table S3: Covariate data sources. Table S4: National HIV mortality rates among men and womenfwil. [file 12916_2020_1876_MOESM1_ESM.docx]

Supplemental Appendix: Mapping HIV mortality in six Latin American countries with incomplete vital registration systems

Table of Contents

[Supplemental Methods 3](#_Toc58333173)

[Vital registration completeness 3](#_Toc58333174)

[Underlying geographic variation in VR completeness 3](#_Toc58333175)

[Calibrating to national VR completeness by age group 5](#_Toc58333176)

[Completeness draws for Brazil and Mexico 7](#_Toc58333177)

[Prior specification 7](#_Toc58333178)

[Statistical model 8](#_Toc58333179)

[References 10](#_Toc58333180)

[Compliance with the Guidelines for Accurate and Transparent Health Estimates Reporting (GATHER) 11](#_Toc58333181)

[Supplemental Figures 13](#_Toc58333182)

[Figure S1: Analytical process overview 13](#_Toc58333183)

[Figure S2: Analytical process for VR data 14](#_Toc58333184)

[Figure S3: Analytical process for VR completeness priors 15](#_Toc58333185)

[Figure S4: Model alignment with GBD, Brazil 16](#_Toc58333186)

[Figure S5: Model alignment with GBD, Colombia 17](#_Toc58333187)

[Figure S6: Model alignment with GBD, Costa Rica 18](#_Toc58333188)

[Figure S7: Model alignment with GBD, Ecuador 19](#_Toc58333189)

[Figure S8: Model alignment with GBD, Guatemala 20](#_Toc58333190)

[Figure S9: Model alignment with GBD, Mexico 21](#_Toc58333191)

[Figure S10: Mean and uncertainty in estimated HIV mortality in Brazil, 2017 22](#_Toc58333192)

[Figure S11: Mean and uncertainty in estimated HIV mortality in Colombia, 2017 23](#_Toc58333193)

[Figure S12: Mean and uncertainty in estimated HIV mortality in Costa Rica, 2016 24](#_Toc58333194)

[Figure S13: Mean and uncertainty in estimated HIV mortality in Ecuador, 2014 25](#_Toc58333195)

[Figure S14: Mean and uncertainty in estimated HIV mortality in Guatemala, 2017 26](#_Toc58333196)

[Figure S15: Mean and uncertainty in estimated HIV mortality in Mexico, 2017 27](#_Toc58333197)

[Figure S16: Estimated HIV mortality in Brazil by age group, 2017 28](#_Toc58333198)

[Figure S17: Estimated HIV mortality in Colombia by age group, 2017 29](#_Toc58333199)

[Figure S18: Estimated HIV mortality in Costa Rica by age group, 2016 30](#_Toc58333200)

[Figure S19: Estimated HIV mortality in Ecuador by age group, 2014 31](#_Toc58333201)

[Figure S20: Estimated HIV mortality in Guatemala by age group, 2017 32](#_Toc58333202)

[Figure S21: Estimated HIV mortality in Mexico by age group, 2017 33](#_Toc58333203)

[Supplemental tables 34](#_Toc58333204)

[Table S1: Merged municipalities by country to form stable geographical units 34](#_Toc58333205)

[Table S2: Vital Registration data 37](#_Toc58333206)

[Table S3: Covariate data sources 39](#_Toc58333207)

[Table S4: National HIV mortality rates among men and women 40](#_Toc58333208)

# Supplemental Methods

## Vital registration completeness

We use a Bayesian hierarchical modelling framework to account for VR systems that vary in completeness by municipality and over time (Figure S1). Our methods expand upon a similar procedure developed in Brazil for estimating life expectancy [1], where a Bayesian framework bypasses a lack of identifiability between the mortality rate and completeness estimate by incorporating an informed prior on the VR completeness. In this analysis, we incorporate information from the GBD [2] on subnational (for Brazil and Mexico) and national VR completeness (for remaining countries) as well as geographic patterns in under-5 VR completeness from past analyses [3] to generate priors on municipality-level VR coverage by two age groups (<15 year-old’s and 15+) and year (Figure S3).

In the present analysis, we model different levels of VR completeness in children and adolescents under 15 years (<15) and for adults ages 15 years and over (15+). We model these age groups separately based on the available national VR completeness estimated in GBD and established literature and expert opinion [4]. We do not model VR completeness for adults if GBD completeness estimates for adults exceeds 95% in all years of available VR (Costa Rica and Colombia). Similarly, we do not model under-15 VR completeness if GBD estimates of completeness is greater than 90% in all years of VR data (Costa Rica, Guatemala, Mexico). We therefore model adult completeness in Ecuador, Guatemala, Mexico, and Brazil, and model under-15 completeness in Ecuador, Colombia, and Brazil.

### Underlying geographic variation in VR completeness

In order to build priors on geographic variation in VR completeness, we used the underlying geographic variation in completeness in under-5 mortality. We estimated VR completeness in under-5 mortality by comparing the estimated number of under-5 deaths in each municipality from previous analyses [3], where they exist, to the reported number of under-5 deaths from VR data. Previous research produced estimates of under-5 mortality in Ecuador, Colombia, and Guatemala that do not rely on vital registration data and produced 1,000 draws of the number of deaths at the 5 x 5-km level [3]. In these three countries, we used these estimates to generate underlying geographic variation in VR completeness. In Mexico and Brazil, we proceed with a slightly different methodology that leverages state-level estimates of completeness produced by GBD and that is described below.

To generate estimates of underlying geographic variation in VR completeness in Ecuador, Colombia, and Guatemala, we first aggregated estimates of under-5 mortality from the 5 x 5-km grid cell level to each municipality at the draw level by year, such that we derived 1,000 draws of the number of under-5 deaths in each area $j$ and year $t$. We aggregated these estimates using the same method as our aggregation of covariates and population—we intersected each grid cell with the municipality-level shapefile to determine what fraction of the area of each grid cell fell within each municipality. For cells split across multiple units, we allocated the number of under-5 deaths in proportion to area. These estimates denote the expected number of deaths in the under-5 age group used to inform the denominator for our initial VR completeness estimates $\pi_{j}^{*}$.

We use the number of reported VR deaths in each area for children under 5 as the numerator for our initial VR completeness estimates $\pi_{j}^{*}$. Due to stochastic variation from year to year in the total number of deaths by area, especially in areas with low child populations, we aggregated VR deaths over all reported years to smooth the number of deaths over time. Nonetheless, after combining child VR deaths across all years in a given area, in some countries there are still areas that report zero deaths. Given that we do not believe completeness is zero in these areas and this likely represents stochastic noise, we used a simple spatial smoothing model to derive more robust estimates of reported under-5 deaths. The spatial smoothing model is outlined below:

$$d_{j} \sim Poisson\left( E_{j}{\cdot e}^{\beta_{0+ S_{j}+ \epsilon_{j}}} \right)$$

$$\epsilon_{j} \sim N\left( 0, \sigma_{\varepsilon}^{2} \right)$$

$$S_{j} \sim ICAR\left( 0, \sigma_{S}^{2} \right)$$

Where $d_{j}$ denoted under-5 deaths in a municipality across all years of available VR data, $E_{j}$ represented the under-5 population summed over all years of available VR data, and $e^{\beta_{0+ S_{j}+ \epsilon_{j}}}$ represented an estimate of the underlying mortality rate—a linear combination in log-space of an intercept $\beta_{0}$, spatially structured random effect $S_{j}$and the unstructured random effect $\epsilon_{j}$. The spatially structured random effect $S_{j}$ is an intrinsic conditional autoregressive model (ICAR) model [6]$.$ The model was fit in R-INLA [5] using a variation of the Besag, York and Mollié (BYM) model [6] to “borrow strength” from the geographic pattern in reported VR deaths while still allowing for non-spatially structured variation. We used first-order queen contiguity of the spatial units to form the graph for the spatial model. We chose this model over the classic BYM model because it parameterizes the relationship between the spatially structured random effect $S_{j}$and the unstructured random effect $\epsilon_{j}$in terms of two hyperparameters: $\tau$ which is the marginal precision and $\varphi$ which is the portion of the marginal variance described by the spatially structured random effect, which improves the interpretability of the hyperparameters. We used the uninformative default penalized complexity priors [6, 7] available in INLA for these hyperparameters:

$$\varphi=PC\left( 0.5, 0.5 \right)$$

$$\tau=PC\left( 1, 0.01 \right)$$

In the first case, this prior indicates a 50% probability that 50% or more of the variation is spatially autocorrelated. In the second, this prior indicates a 1% chance that the log precision is less than 1. After fitting the model, we calculate the smoothed number of VR under-5 deaths by using the posterior mean estimate of the mortality rate for each area $j$ and multiplying by the sum of the under-5 population over all years. We produce 1,000 draws ($i$*)* of the underlying completeness $\pi^{*}$for each area $j$:

$$\pi_{j,i}^{*} = \frac{{VR deaths}_{j}}{{U5M deaths}_{j, i}}$$

There are certain areas where ${VR death}_{j}> {U5M deaths}_{j,i}$ and underlying completeness estimates are above 1. Given that we have no reason to believe certain areas are over-reporting deaths, we truncated completeness to either the 99^th^ percentile of completeness draws in that municipality or 0.99, whichever is greater.

### Calibrating to national VR completeness by age group

The methods outlined above produced estimates of subnational geographic variation in VR completeness by municipality in Ecuador, Colombia, and Guatemala, but this variation is not specific to year or age group. We proceed with two different frameworks, one for adult VR completeness estimates and one for under-15 completeness estimates. For both under-15 and adults, we rescale the municipality-level completeness estimates such that the death-weighted aggregation matches the GBD national VR completeness estimates.

For national adult VR completeness, GBD produces 1,000 draws ($i$) of completeness for each country and year $t$, $\Pi_{t,i}$. We rescale our initial estimates of municipality-level VR completeness, $\pi_{j,i}^{*}$, at the draw level such that the expected number of true deaths among adults in each area $j$ and year $t$, calculated as the number of reported adult VR deaths $d_{j,t}^{adult}$divided by completeness $\pi_{j,i}^{*}$ is equal to the total number of expected national deaths by year $D_{t}^{adult}$. The total number of expected national deaths $D_{t}^{adult}$ is calculated as the sum of all municipality-level adult VR deaths $D_{t}^{adult} = \sum_{j} d_{j,t}^{adult}$ divided by the national GBD completeness $\Pi_{t,i}$. We rescale the municipality-level completeness estimates at the draw level in logit space to ensure completeness remains between zero and one while scaling the expected number of deaths to GBD by adding an adjustment factor $c_{t,i}^{adult}$ as represented in the equation below for each country:

$$\sum_{j} \left( \frac{d_{j,t}^{adult}}{{\mathrm{logit}^{-1}(logit(\pi}_{j,i}^{*}+c_{t,i}^{adult}))} \right)= \frac{D_{t}^{adult}}{\Pi_{t,i}}$$

$$\sum_{j} d_{j,t}^{adult}=D_{t}^{adult}$$

We calculated and applied 1,000 draws of the adjustment factor $c_{t,i}^{adult}$ to each municipality-draw of the initial completeness in year $t$ to produce 1,000 draws of initial completeness for each municipality and year: $\pi_{j,t,i}^{\mathrm{adult}} = {\mathrm{logit}^{-1}(logit(\pi}_{j,i}^{*}+c_{t,i}^{adult}))$.

  For under-15 VR completeness, we undertook a different approach given that GBD does not produce draws of child completeness. In this case, for each country and year $t$ we pulled 1,000 draws of the estimated under-15 all-cause deaths from the GBD,$D_{t}^{under15}$. We then rescaled the expected number under-15 deaths in municipality $j$ and year $t$ to equal to the estimated number of under-15 deaths from GBD by applying an adjustment factor $c_{t,i}^{under15}$ to each municipality in logit space:

$$\sum_{j} \frac{d_{j,t}^{under15}}{{\mathrm{logit}^{-1}(logit(\pi}_{j,i}^{*}+c_{t,i}^{under15} ))}= D_{t,i}^{under15}$$

We calculated and applied 1,000 draws of the adjustment factor $c_{t,i}^{under15}$ to each municipality-draw of the initial completeness in year $t$ to produce 1,000 draws of initial completeness for each municipality and year: $\pi_{j,t,i}^{under15}= {\mathrm{logit}^{-1}(logit(\pi}_{j,i}^{*}+c_{t,i}^{under15`}))$.

### Completeness draws for Brazil and Mexico

For Brazil and Mexico, we leverage state-level estimates of VR completeness for adults and children produced by GBD [2]. For state-level adult VR completeness, GBD produces 1,000 draws ($i$) of completeness for each state $J$ and year $t$, $\Pi_{J,t,i}$.These estimates of adult completeness at the state level are modelled directly in our small area estimation framework, where each municipality that nests within a state is assumed to follow the same prior and contributes to the same posterior level of VR completeness.

For under-15 completeness in Brazil, given that GBD does not produce draw-level completeness, we extracted 1,000 draws of estimated under-15 all-cause deaths for each state $J$ and year $t$: $D_{J,t,i}$. We then calculate draws of completeness by taking the ratio of the reported all-cause deaths for each state $J$ and year $t$ from VR data and $D_{J,t,i}$:

$$\pi_{J,t,i}^{under15}=\frac{{VR deaths}_{J,t}}{D_{J,t,i}}$$

In a small number of state draws, completeness estimates are greater than 1 and these are truncated to 0.99. These estimates of under-15 completeness at the state level are modelled directly in our small area estimation framework, where each municipality that nests within a state inherits the same prior and contributes to the posterior for VR completeness.

### Prior specification

The processes outlined above generate draws of both adult and under-15 completeness for each municipality (Ecuador, Colombia, Guatemala) or state (Brazil and Mexico) by year. To include these informed priors in our modelling framework, we characterized the distribution by fitting to a logit-normal distribution using maximum likelihood estimation. For area-municipality-years where all draws were truncated at 0.99, we fit the model with a mean of 0.99 and a standard deviation of 0.01 in logit space.

## Statistical model

We fit the following hierarchical generalized linear model for VR data, building on a model developed in prior modelling studies [8, 9]

$$D_{j,t,a}\sim Poisson\left( m_{j,t,a}\cdot\pi_{k,t,a^{*}}\cdot P_{j,t,a} \right)$$

$$\gamma_{1,a,t}\sim LCAR:LCAR\left( \sigma_{1}^{2},\rho_{1,A},\rho_{1,T} \right)$$

$$\gamma_{2,j}\sim\mathrm{LCAR}\left( \sigma_{2}^{2},\rho_{2} \right)$$

$$\gamma_{3,j}\sim\mathrm{LCAR}\left( \sigma_{3}^{2},\rho_{3} \right)$$

$$\gamma_{4,j}\sim\mathrm{LCAR}\left( \sigma_{4}^{2},\rho_{4} \right)$$

$$\gamma_{5,j,t}\sim N\left( {0, \sigma}_{5}^{2} \right)$$

$$\gamma_{6,j,a}\sim N\left( {0, \sigma}_{6}^{2} \right)$$

$$\frac{1}{\sigma_{i}^{2}} \sim\mathrm{Gamma}\left( 1, 1000 \right) \mathrm{for} i\in1, 2, 3, 4, 5, 6$$

$$\mathrm{logit}\left( \rho_{i} \right) \sim\mathrm{Normal}\left( 0, 1.5 \right) \mathrm{for} i\in1A,1T, 2, 3, 4$$

where $D_{j,t,a}$ represents the number of HIV deaths in municipality *j*, year *t,* and age group *a;* $m_{j,t,a}$ is the mortality rate in municipality *j*, year *t,* and age group *a*; $\pi_{k,t,a^{*}}$ is the VR completeness in municipality (Colombia, Ecuador, and Guatemala) or state (Brazil and Mexico) *j*, year *t,* and completeness age group $a^{*}$ (<15, 15+); $P_{j,t,a}$ is the population in municipality *j*, year *t,* and age group *a*; $\beta_{0}$ is the intercept; $\beta_{1}\cdot X_{j}$ is the vector of covariates and associated regression coefficients; $\gamma_{1,a,t}$ describes the overall age-time pattern; $\gamma_{2,j}$ describes spatial patterns that persist over age and time, $\gamma_{3,j}\cdot t$ describes area-specific deviations from the overall time pattern; $\gamma_{4,j}\cdot a$ describes area-specific deviations from the overall age pattern; $\gamma_{5,j,t}$and $\gamma_{6,j,a}$allow for area-specific non-linear deviations from the overall time and age patterns, respectively.

VR completeness is incorporated into the data generating model, and logit-normal priors on $\pi_{k,t,a^{*}}$ fit on empirical data as described above allow the model to distinguish between mortality rate $m_{j,t,a}$ and the VR completeness. Random effects $\gamma_{1,a,t},\gamma_{2,j},\gamma_{3,j},\gamma_{4,j}$ were assigned a Leroux conditional autoregressive prior (LCAR) [10]. The full conditional distribution can be described by:

$$\gamma_{i}|\gamma_{k\sim i}, \sigma^{2},\rho\sim\mathrm{Normal}\left( \frac{\rho\sum_{k\sim i} \gamma_{k}}{n_{i}\cdot\rho+1-\rho},\frac{\sigma^{2}}{n_{i}\cdot\rho+1-\rho} \right)$$

where: $k \sim i$ denotes the set of $i$’s “neighbors” (for spatial terms, municipalities that share a border; for temporal/age terms, adjacent years/age groups); $n_{j}$ is the number of neighbors in $k \sim i$; $\sigma^{2}$ is the variance parameter; and $\rho$ is the correlation parameter. These random effects allow for additional variation across space, time, and age that is not explained by the covariates. For each of the random effects, the variance ($\sigma^{2}$) denotes the amount of variation, while the correlation ($\rho)$ determines how much smoothing takes place, $\rho$ ranged 0 to 1 with higher values indicating greater spatial smoothness. We assigned Gamma(0, 1000) hyperpriors for the precision of each random effect and Normal(0, 1.5) hyperpriors for the logit-transform of the correlation parameters. The random effects $\gamma_{5,j,t}$ and $\gamma_{6,j,a}$ were assumed to follow independent mean-zero normal distributions.

We model $\gamma_{1}$ as an interaction between two conditional autoregressive (LCAR) distributions as defined above for age and time, respectively. This was specified according to the procedure described by Knorr-Held (i.e., a ‘Type IV’ interaction) [11]. This specification allows for smoothing over age group and time simultaneously, such that the level for a given age group and year is informed both by first-order neighbors (i.e., adjacent years in the same age group and adjacent age groups in the same year) as well as second order neighbors (i.e., adjacent years in adjacent age groups). For this distribution there are three hyperparameters: $\sigma^{2}$, which control the overall amount of variation, and $\rho_{1,A}$ and $\rho_{1,T}$which control the smoothness over age and time, respectively.

# References

1. Schmertmann CP, Gonzaga MR. Bayesian Estimation of Age-Specific Mortality and Life Expectancy for Small Areas With Defective Vital Records. Demography. 2018;55:1363–88.

2. Roth GA, Abate D, Abate KH, Abay SM, Abbafati C, Abbasi N, et al. Global, regional, and national age-sex-specific mortality for 282 causes of death in 195 countries and territories, 1980–2017: a systematic analysis for the Global Burden of Disease Study 2017. The Lancet. 2018;392:1736–88.

3. Burstein R, Henry N, Collison M, Marczak L, Sligar A, Watson S, et al. Mapping 123 million neonatal, infant and child deaths between 2000 and 2017. Nature. 2019;574:353–358.

4. Målqvist M, Eriksson L, Nga N, Fagerland L, Hoa D, Ewald U, et al. Unreported births and deaths, a severe obstacle for improved neonatal survival in low-income countries; a population based study. BMC international health and human rights. 2008;8:4.

5. The R-INLA project. R-INLA. 2019. http://www.r-inla.org/. Accessed 25 Jul 2019.

6. Riebler A, Sørbye S, Simpson D, Rue H. An intuitive Bayesian spatial model for disease mapping that accounts for scaling. 2015.

7. Fuglstad G-A, Simpson D, Lindgren F, Rue H. Constructing Priors that Penalize the Complexity of Gaussian Random Fields. Journal of the American Statistical Association. 2018.

8. Ross J, Henry N, Dwyer-Lindgren L, Lobo A, Marinho de Souza MDF, Biehl M, et al. Progress toward eliminating TB and HIV deaths in Brazil, 2001-2015: A spatial assessment. BMC Medicine. 2018;16.

9. Dwyer-Lindgren L, Bertozzi-Villa A, Stubbs RW, Morozoff C, Kutz MJ, Huynh C, et al. US County-Level Trends in Mortality Rates for Major Causes of Death, 1980-2014. JAMA. 2016;316:2385–401.

10. Leroux BG, Lei X, Breslow N. Estimation of Disease Rates in Small Areas: A new Mixed Model for Spatial Dependence. In: Halloran ME, Berry D, editors. Statistical Models in Epidemiology, the Environment, and Clinical Trials. New York, NY: Springer; 2000. p. 179–91.

11. Knorr-Held L. Bayesian modelling of inseparable space-time variation in disease risk. Stat Med. 2000;19:2555–67.

12. Kristensen K, Nielsen A, Berg CW, Skaug H, Bell BM. TMB: Automatic Differentiation and Laplace Approximation. Journal of Statistical Software. 2016;70:1–21.

13. R Core Team. R: a language and environment for statistical computing. Vienna, Austria: Foundation for Statistical Computing; 2015.

# Compliance with the Guidelines for Accurate and Transparent Health Estimates Reporting (GATHER)

| Item # | Checklist item | Description of Compliance |
| --- | --- | --- |
| Objectives and funding | | |
| 1 | Define the indicator(s), populations (including age, sex, and geographical entities), and time period(s) for which estimates were made. | Manuscript: Background, Methods |
| 2 | List the funding sources for the work. | Manuscript: Declarations (Funding) |
| Data Inputs | | |
| *For all data inputs from multiple sources that are synthesised as part of the study:* | | |
| 3 | Describe how the data were identified and how the data were accessed. | Manuscript: Methods |
| 4 | Specify the inclusion and exclusion criteria. Identify all ad-hoc exclusions. | Manuscript: Methods |
| 5 | Provide information on all included data sources and their main characteristics. For each data source used, report reference information or contact name/institution, population represented, data collection method, year(s) of data collection, sex and age range, diagnostic criteria or measurement method, and sample size, as relevant. | Supplemental Tables S1 and S2 |
| 6 | Identify and describe any categories of input data that have potentially important biases (e.g., based on characteristics listed in item 5). | Manuscript: Discussion (Limitations) |
| *For data inputs that contribute to the analysis but were not synthesised as part of the study:* | | |
| 7 | Describe and give sources for any other data inputs. | Manuscript: Methods; Supplemental Table S3 |
| *For all data inputs:* | | |
| 8 | Provide all data inputs in a file format from which data can be efficiently extracted (e.g., a spreadsheet rather than a PDF), including all relevant meta-data listed in item 5. For any data inputs that cannot be shared because of ethical or legal reasons, such as third-party ownership, provide a contact name or the name of the institution that retains the right to the data. | Available through GHDx: <http://ghdx.healthdata.org/record/ihme-data/latin-america-hiv-mortality-estimates-2000-2017> |
| Data analysis | | |
| 9 | Provide a conceptual overview of the data analysis method. A diagram may be helpful. | Manuscript: Methods; Figure S1-S3 |
| 10 | Provide a detailed description of all steps of the analysis, including mathematical formulae. This description should cover, as relevant, data cleaning, data pre-processing, data adjustments and weighting of data sources, and mathematical or statistical model(s). | Manuscript: Methods |
| 11 | Describe how candidate models were evaluated and how the final model(s) were selected. | Manuscript: Methods |
| 12 | Provide the results of an evaluation of model performance, if done, as well as the results of any relevant sensitivity analysis. | Manuscript: Methods |
| 13 | Describe methods for calculating uncertainty of the estimates. State which sources of uncertainty were, and were not, accounted for in the uncertainty analysis. | Manuscript: Methods |
| 14 | State how analytic or statistical source code used to generate estimates can be accessed. | Available through GHDx: <http://ghdx.healthdata.org/record/ihme-data/latin-america-hiv-mortality-estimates-2000-2017> |
| Results and Discussion | | |
| 15 | Provide published estimates in a file format from which data can be efficiently extracted. | Available through GHDx: <http://ghdx.healthdata.org/record/ihme-data/latin-america-hiv-mortality-estimates-2000-2017> |
| 16 | Report a quantitative measure of the uncertainty of the estimates (e.g., uncertainty intervals). | Manuscript: Results |
| 17 | Interpret results in light of existing evidence. If updating a previous set of estimates, describe the reasons for changes in estimates. | Manuscript: Discussion |
| 18 | Discuss limitations of the estimates. Include a discussion of any modelling assumptions or data limitations that affect interpretation of the estimates. | Manuscript: Discussion |

# Supplemental Figures

## Figure S1: Analytical process overview


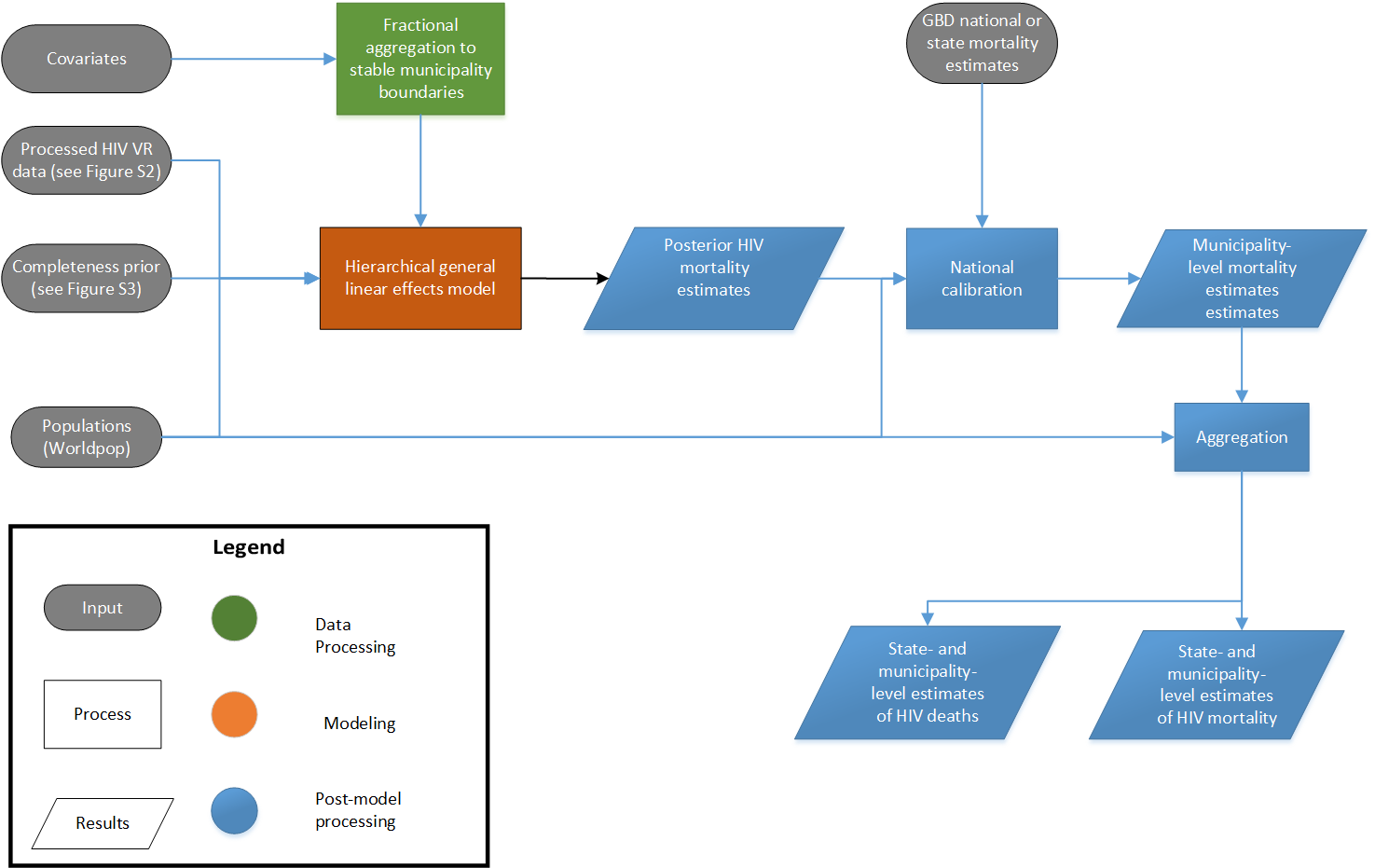


**Figure S1: Analytical process overview.** The process used to produce HIV mortality estimates by age, sex, year, and municipality involved three main parts. In the data processing steps (green) data were identified, extracted and prepared for use in the HIV mortality model. In the modeling phase (orange) we used data and covariates in a hierarchical linear effects model. In the post-model processing (blue) we calibrated mortality estimates to national GBD 2017 estimates, aggregated mortality estimates to the state level, and calculated the number of HIV deaths.

## Figure S2: Analytical process for VR data


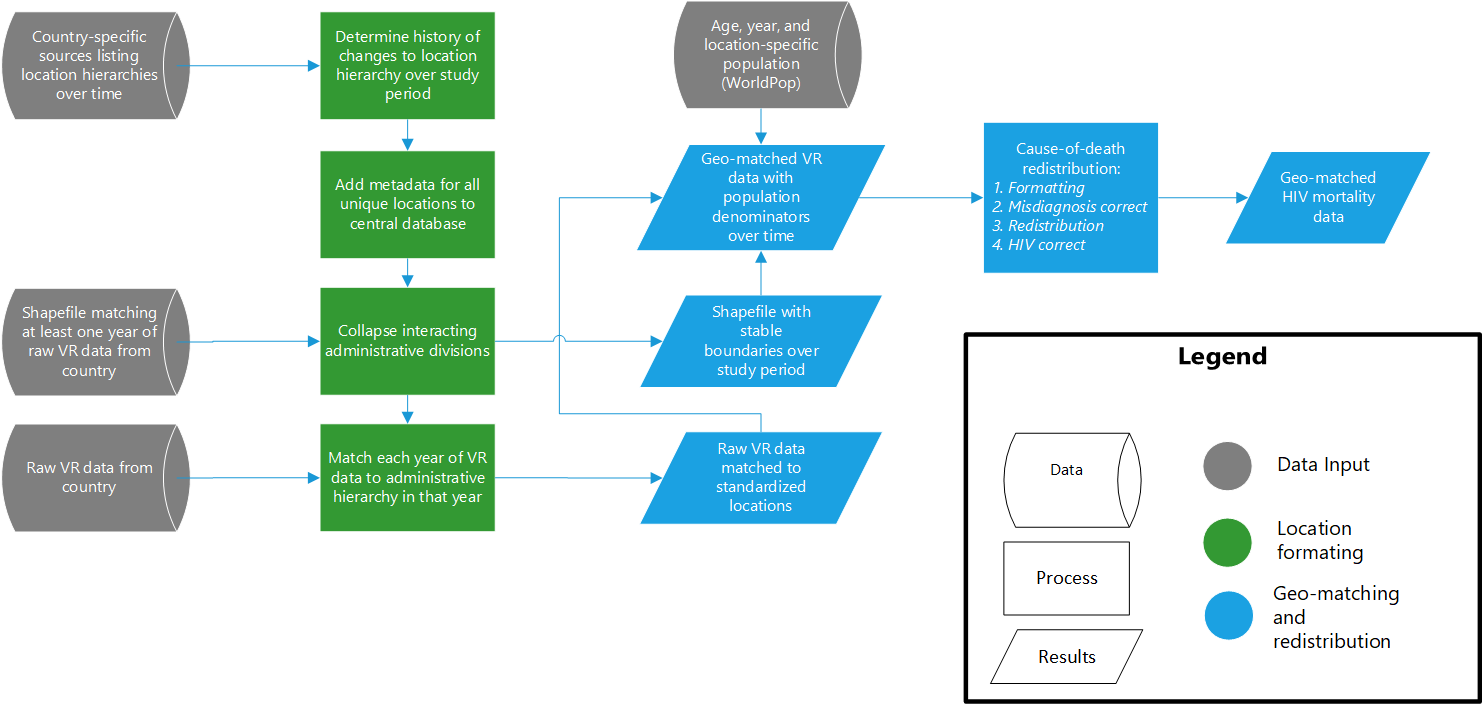


**Figure S2: Analytical process for VR data.**  The process used to process VR data for our analysis consisted of three main parts. In the data input steps (grey) country specific shapefiles and location hierarchies were acquired to match to raw VR data. In the location formatting steps (green) we matched VR data to stable areas over the years of study. In the geo-matching and redistribution phase (blue) we produced a stable shapefile over the years of study and raw VR data was processed using cause of death redistribution as outlined in GBD 2017. At the end of this process, we produced HIV mortality data matched to stable municipalities within each country.

## Figure S3: Analytical process for VR completeness priors


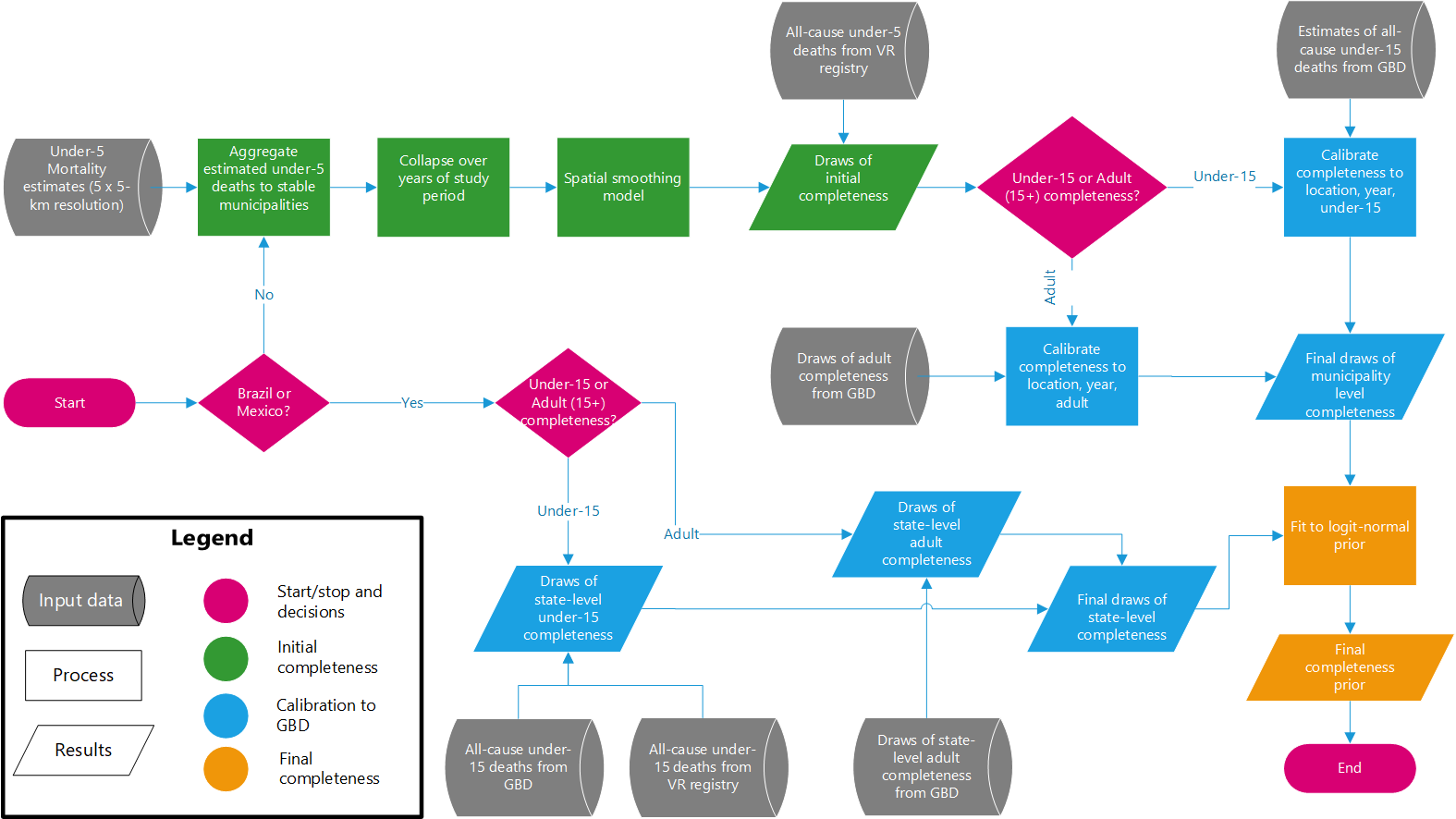


**Figure S3: Analytical process overview for VR completeness priors.** The process used to process VR data for our analysis consisted of three main parts. In the initial completeness steps (green) for Colombia, Ecuador, and Guatemala, draws of initial completeness were produced using under-5 mortality estimates. In the calibration to GBD steps (blue) state- or municipality-level initial completeness estimates were calibrated to GBD 2017 using draws of national adult completeness or under-15 national deaths. In the final completeness steps (orange) a logit-normal prior was fit to draws of completeness to generate a final completeness prior used in the modeling process.

## Figure S4: Model alignment with GBD, Brazil


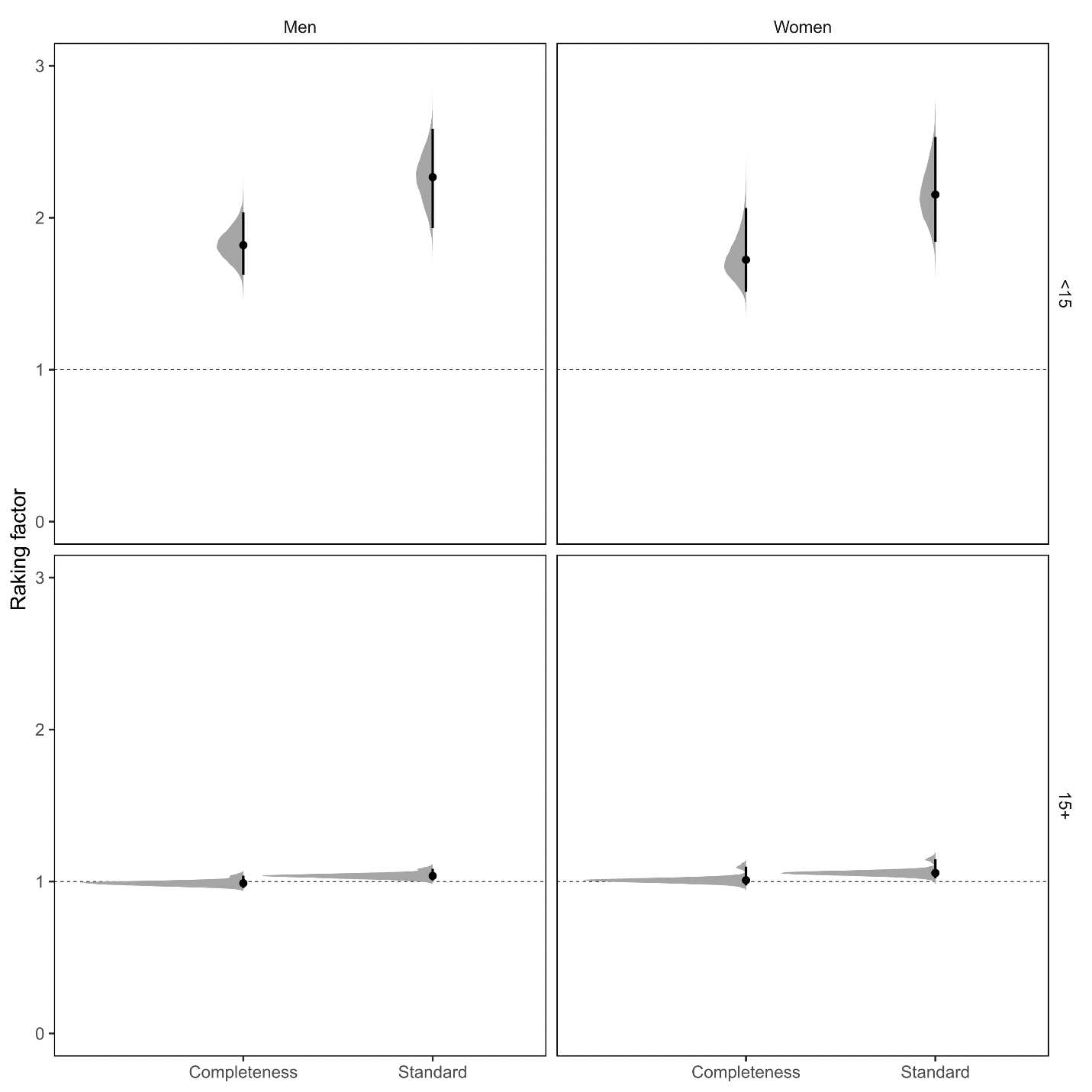


**Figure S4: Model alignment with GBD, Brazil.**  Comparison of the annual ratio of national HIV mortality from GBD and 1,000 draws of annual national HIV mortality in children under-15 (<15) and adults (15+) by sex across the entire range of study (2000 to 2017). The model used in the analysis that includes prior completeness $\pi_{k,t,a^{*}}$ (‘Completeness’) is shown compared to a model without any prior information on completeness (‘Standard’). Each point represents the median of the draws of the raking factor, the bar represents 2.5^th^ and 97.5^th^ quantile of the draws, and the density curve represents the relative frequency of the draws. A raking factor closer to 1 (dotted line) indicates better alignment between model results and GBD estimates.

## Figure S5: Model alignment with GBD, Colombia


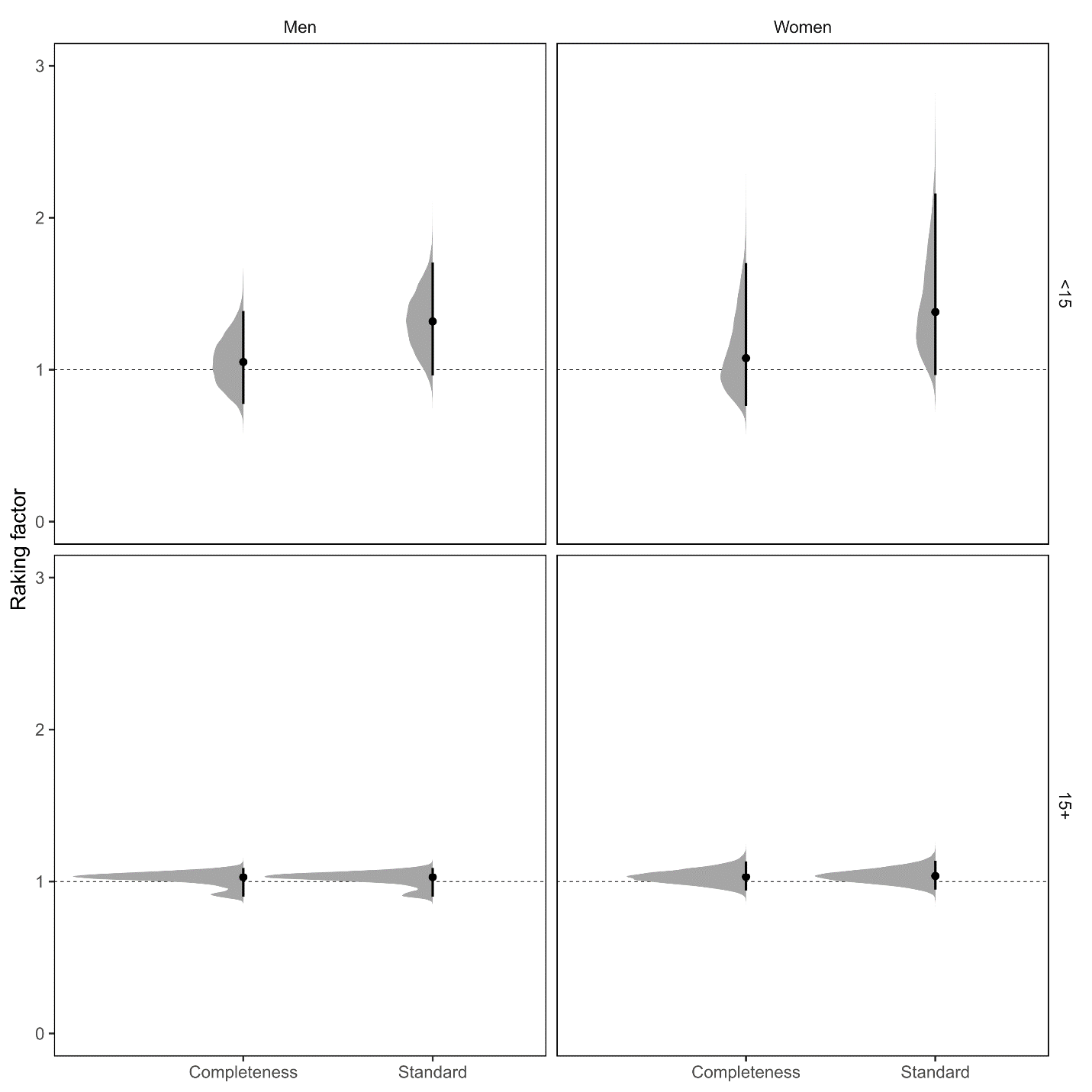


**Figure S5: Model alignment with GBD, Colombia.**  Comparison of the annual ratio of national HIV mortality from GBD and 1,000 draws of annual national HIV mortality in children under-15 (<15) and adults (15+) by sex across the entire range of study (2000 to 2017). The model used in the analysis that includes prior completeness $\pi_{k,t,a^{*}}$ (‘Completeness’) is shown compared to a model without any prior information on completeness (‘Standard’). Each point represents the median of the draws of the raking factor, the bar represents 2.5^th^ and 97.5^th^ quantile of the draws, and the density curve represents the relative frequency of the draws. A raking factor closer to 1 (dotted line) indicates better alignment between model results and GBD estimates.

## Figure S6: Model alignment with GBD, Costa Rica


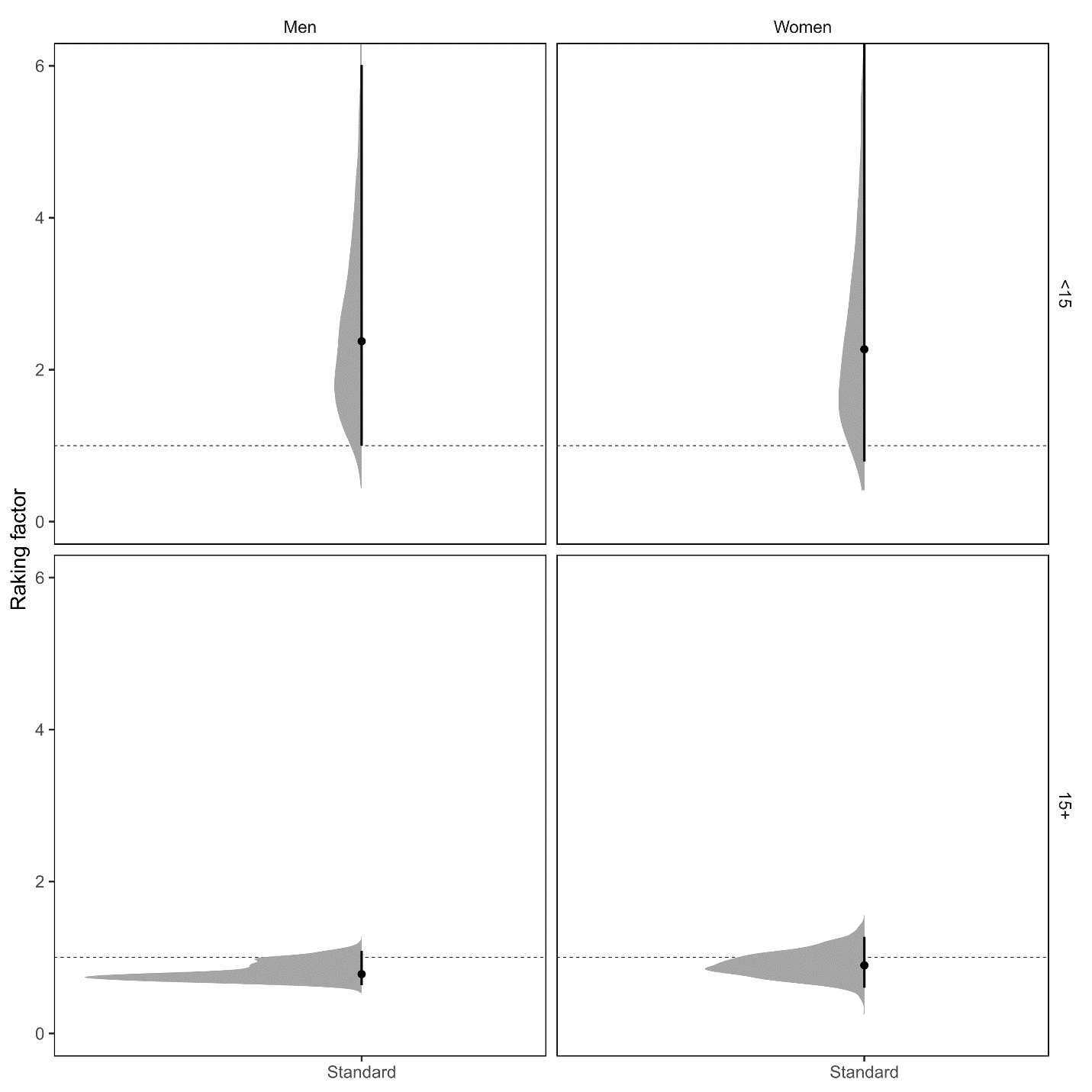


**Figure S6: Model alignment with GBD, Costa Rica.**  Comparison of the annual ratio of national HIV mortality from GBD and 1,000 draws of annual national HIV mortality in children under-15 (<15) and adults (15+) by sex across the entire range of the study (2014 to 2016). We do not model prior completeness for Costa Rica, and we show the final model without any prior information on completeness (‘Standard’). Each point represents the median of the draws of the raking factor, the bar represents 2.5^th^ and 97.5^th^ quantile of the draws, and the density curve represents the relative frequency of the draws. A raking factor closer to 1 (dotted line) indicates better alignment between model results and GBD estimates.

## Figure S7: Model alignment with GBD, Ecuador


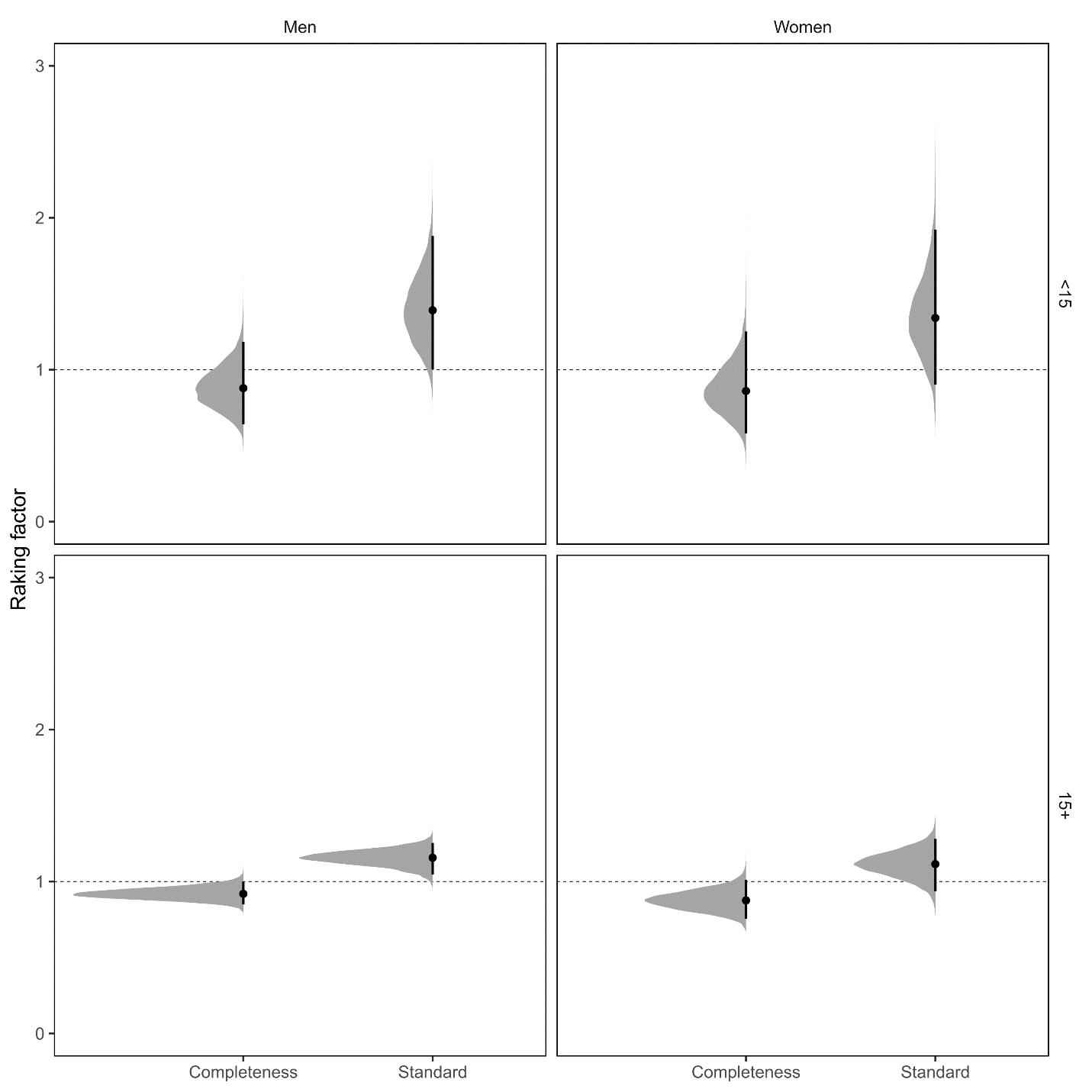


**Figure S7: Model alignment with GBD, Ecuador.**  Comparison of the annual ratio of national HIV mortality from GBD and 1,000 draws of annual national HIV mortality in children under-15 (<15) and adults (15+) by sex across the entire range of study (2004 to 2014). The model used in the analysis that includes prior completeness $\pi_{k,t,a^{*}}$ (‘Completeness’) is shown compared to a model without any prior information on completeness (‘Standard’). Each point represents the median of the draws of the raking factor, the bar represents 2.5^th^ and 97.5^th^ quantile of the draws, and the density curve represents the relative frequency of the draws. A raking factor closer to 1 (dotted line) indicates better alignment between model results and GBD estimates.

## Figure S8: Model alignment with GBD, Guatemala


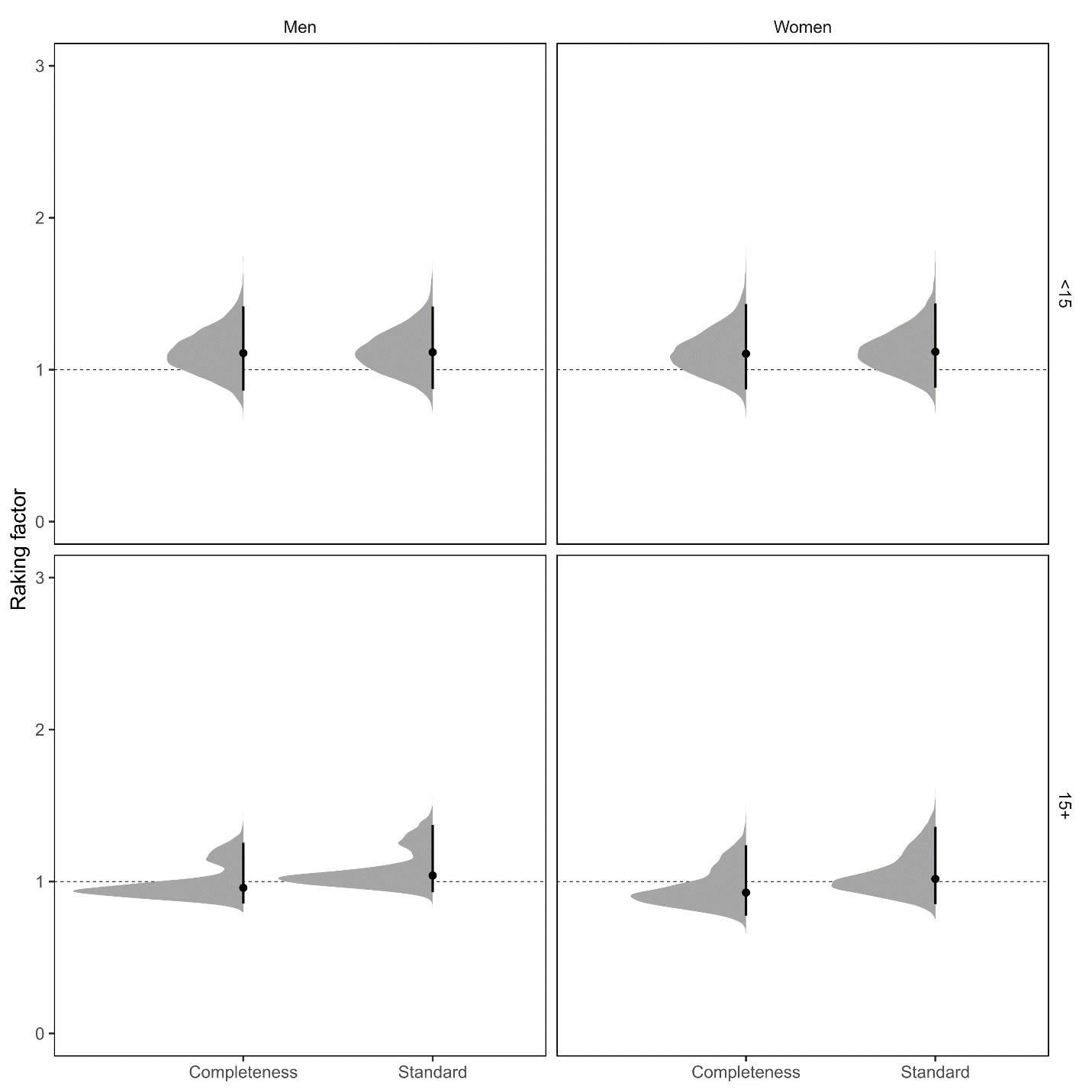


**Figure S8: Model alignment with GBD, Guatemala.**  Comparison of the annual ratio of national HIV mortality from GBD and 1,000 draws of annual national HIV mortality in children under-15 (<15) and adults (15+) by sex across the entire range of study (2009 to 2017). The model used in the analysis that includes prior completeness $\pi_{k,t,a^{*}}$ (‘Completeness’) is shown compared to a model without any prior information on completeness (‘Standard’). Each point represents the median of the draws of the raking factor, the bar represents 2.5^th^ and 97.5^th^ quantile of the draws, and the density curve represents the relative frequency of the draws. A raking factor closer to 1 (dotted line) indicates better alignment between model results and GBD estimates.

## Figure S9: Model alignment with GBD, Mexico


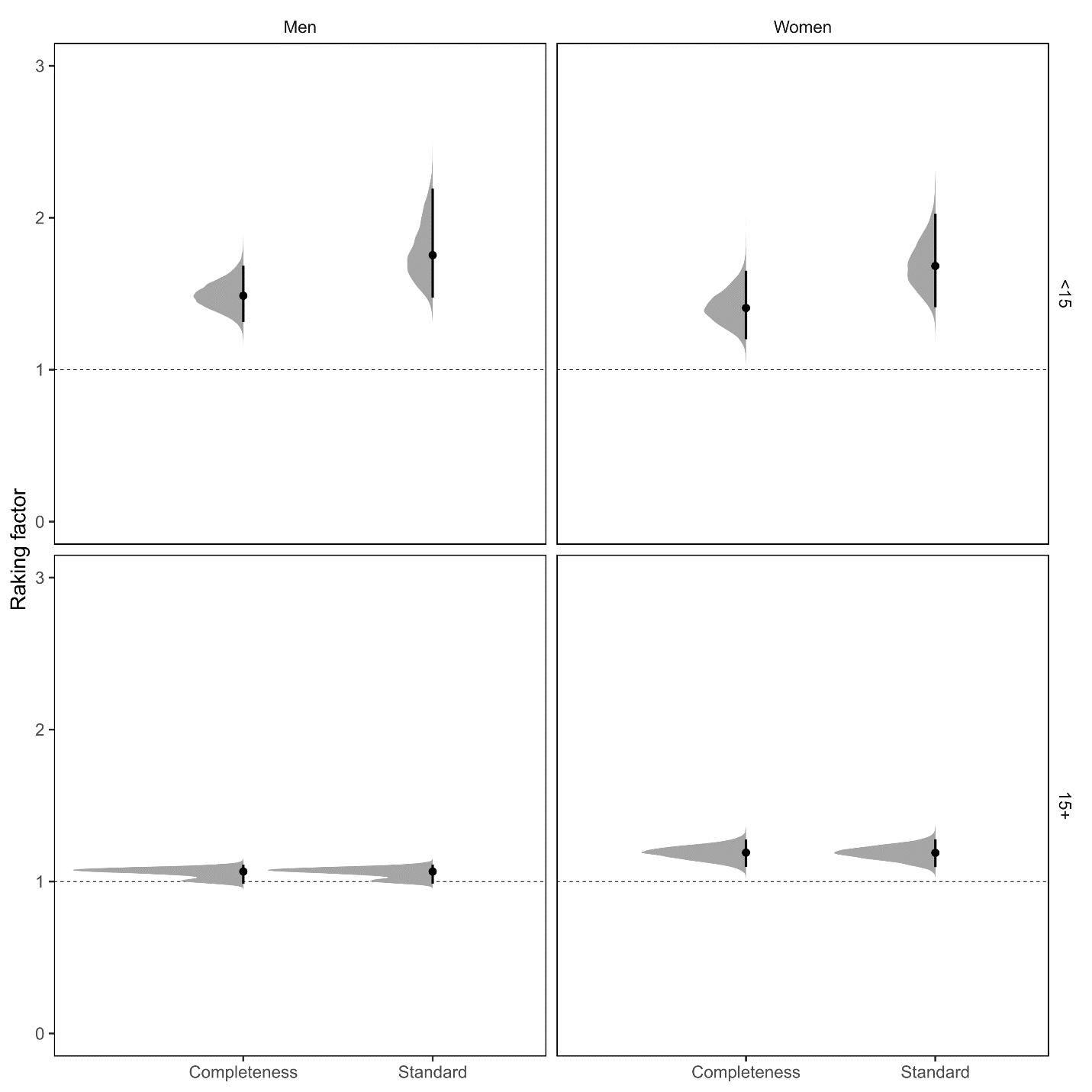


**Figure S9: Model alignment with GBD, Mexico.**  Comparison of the annual ratio of national HIV mortality from GBD and 1,000 draws of annual national HIV mortality in children under-15 (<15) and adults (15+) by sex across the entire range of study (2000 to 2017). The model used in the analysis that includes prior completeness $\pi_{k,t,a^{*}}$ (‘Completeness’) is shown compared to a model without any prior information on completeness (‘Standard’). Each point represents the median of the draws of the raking factor, the bar represents 2.5^th^ and 97.5^th^ quantile of the draws, and the density curve represents the relative frequency of the draws. A raking factor closer to 1 (dotted line) indicates better alignment between model results and GBD estimates.

## Figure S10: Mean and uncertainty in estimated HIV mortality in Brazil, 2017


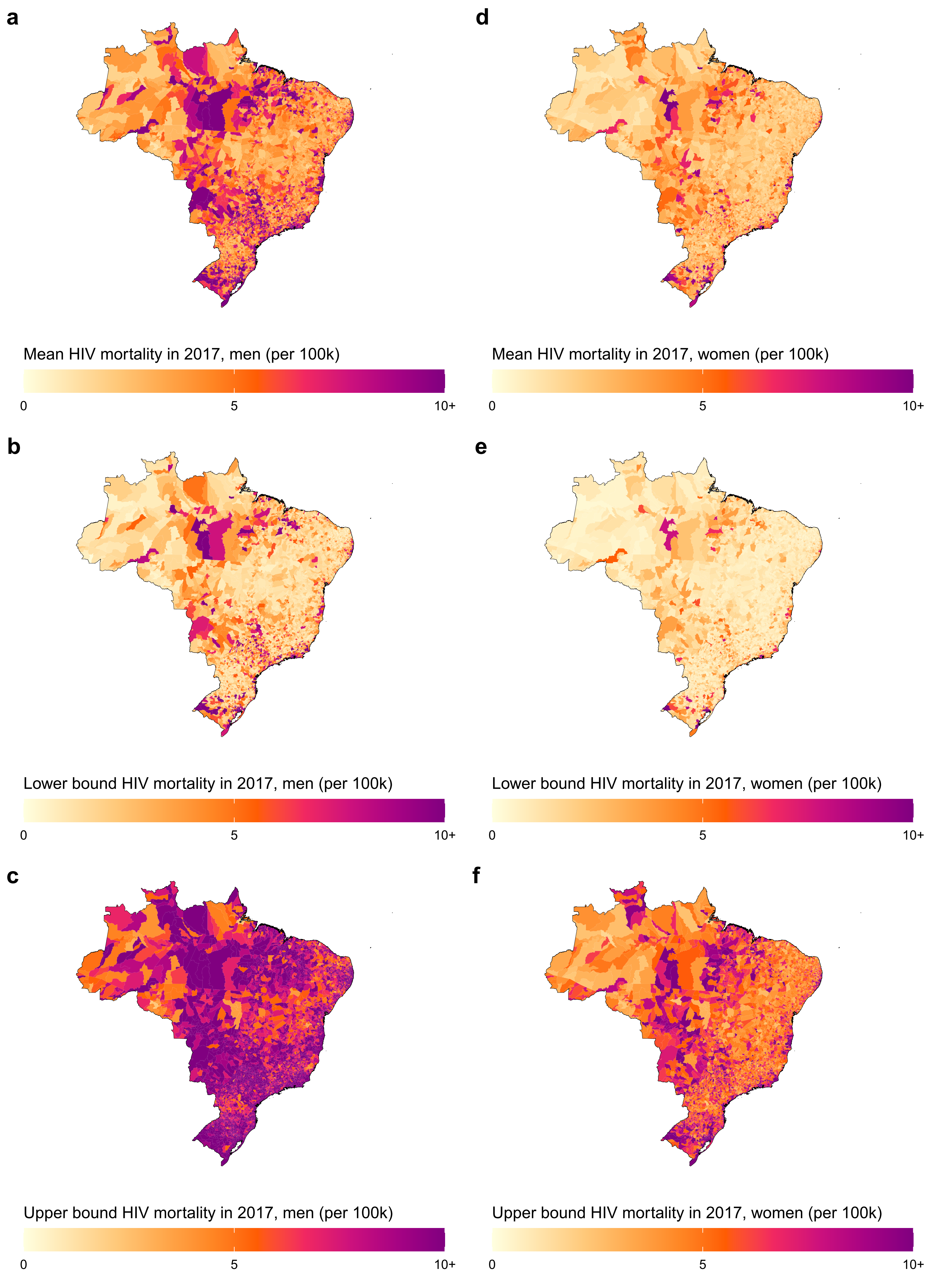


**Figure S10: Mean and uncertainty in estimated HIV mortality in Brazil, 2017.**  Estimated HIV mortality at the municipality level in 2017 in Brazil among men (**a-c**) and women (**d-f**). Mean estimates and lower and upper bounds of the 95% uncertainty intervals are shown in the top, middle, and bottom column, respectively.

## Figure S11: Mean and uncertainty in estimated HIV mortality in Colombia, 2017


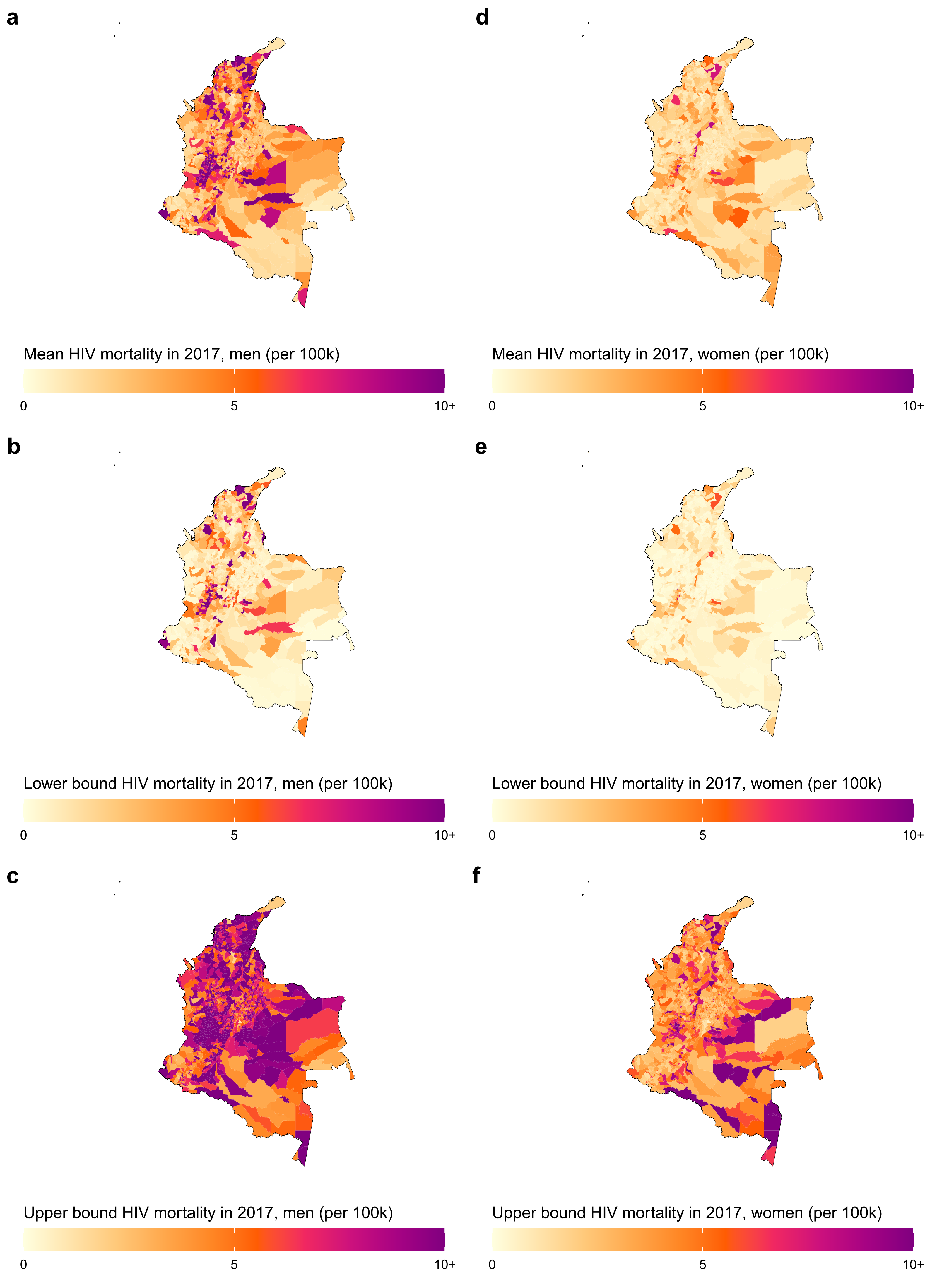


**Figure S11: Mean and uncertainty in estimated HIV mortality in Colombia, 2017.**  Estimated HIV mortality at the municipality level in 2017 in Colombia among men (**a-c**) and women (**d-f**). Mean estimates and lower and upper bounds of the 95% uncertainty intervals are shown in the top, middle, and bottom column, respectively.

## Figure S12: Mean and uncertainty in estimated HIV mortality in Costa Rica, 2016


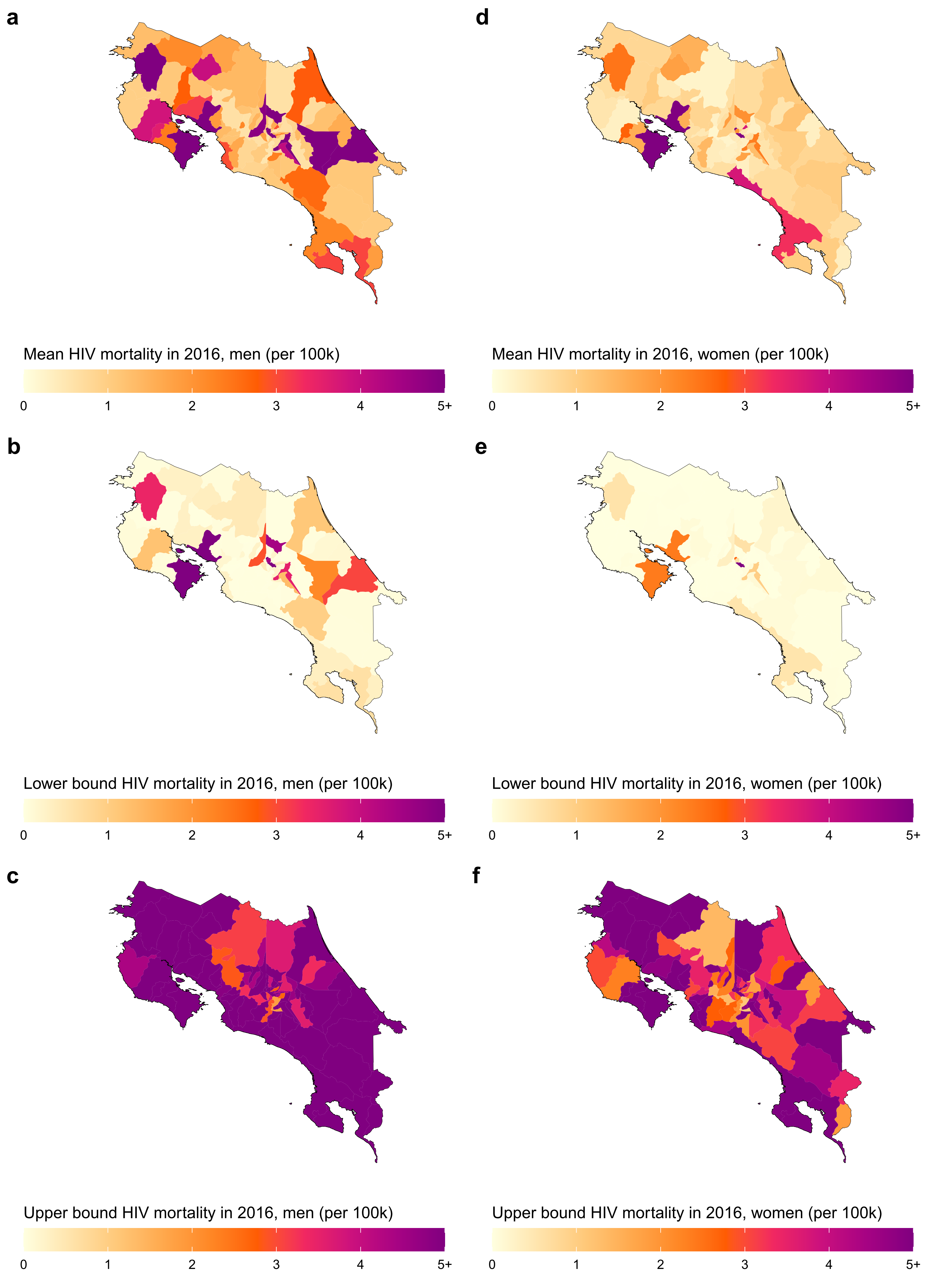


**Figure S12: Mean and uncertainty in estimated HIV mortality in Costa Rica, 2016.**  Estimated HIV mortality at the canton level in 2016 in Costa Rica among men (**a-c**) and women (**d-f**). Mean estimates and lower and upper bounds of the 95% uncertainty intervals are shown in the top, middle, and bottom column, respectively.

## Figure S13: Mean and uncertainty in estimated HIV mortality in Ecuador, 2014


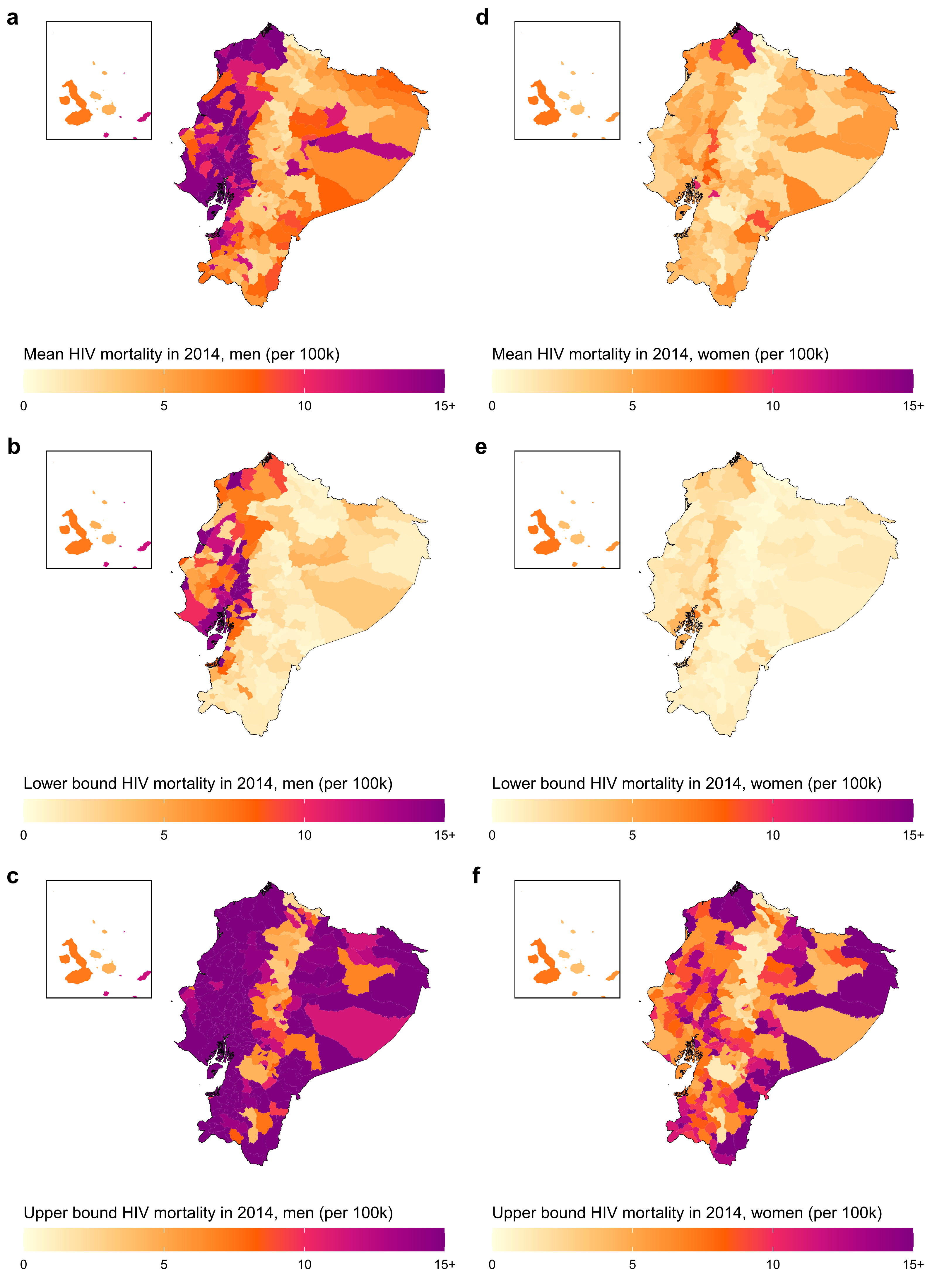


**Figure S13: Mean and uncertainty in estimated HIV mortality in Ecuador, 2014.**  Estimated HIV mortality at the canton level in 2014 in Ecuador among men (**a-c**) and women (**d-f**). Mean estimates and lower and upper bounds of the 95% uncertainty intervals are shown in the top, middle, and bottom column, respectively.

## Figure S14: Mean and uncertainty in estimated HIV mortality in Guatemala, 2017


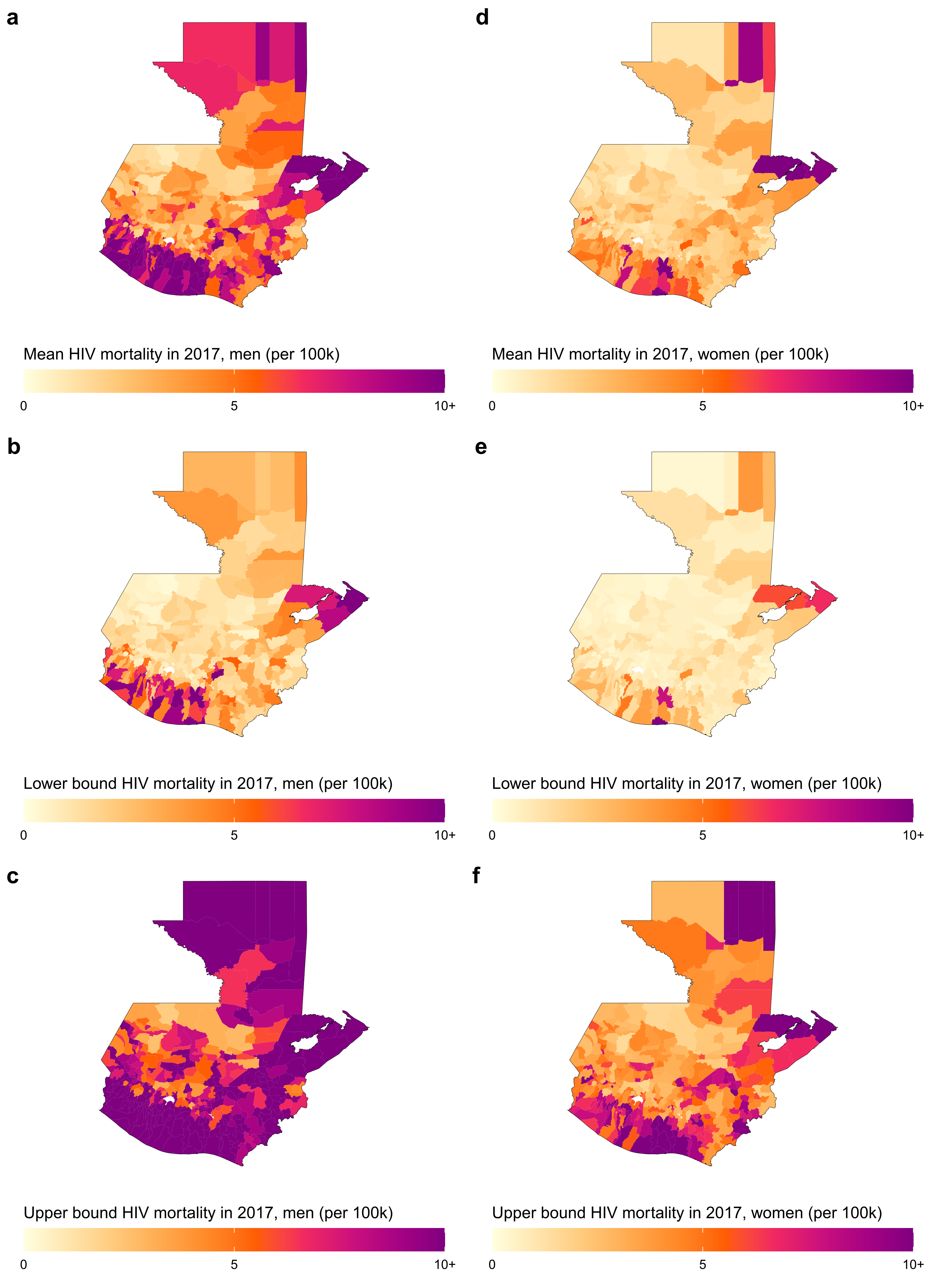


**Figure S14: Mean and uncertainty in estimated HIV mortality in Guatemala, 2017.**  Estimated HIV mortality at the municipality level in 2017 in Guatemala among men (**a-c**) and women (**d-f**). Mean estimates and lower and upper bounds of the 95% uncertainty intervals are shown in the top, middle, and bottom column, respectively.

## Figure S15: Mean and uncertainty in estimated HIV mortality in Mexico, 2017


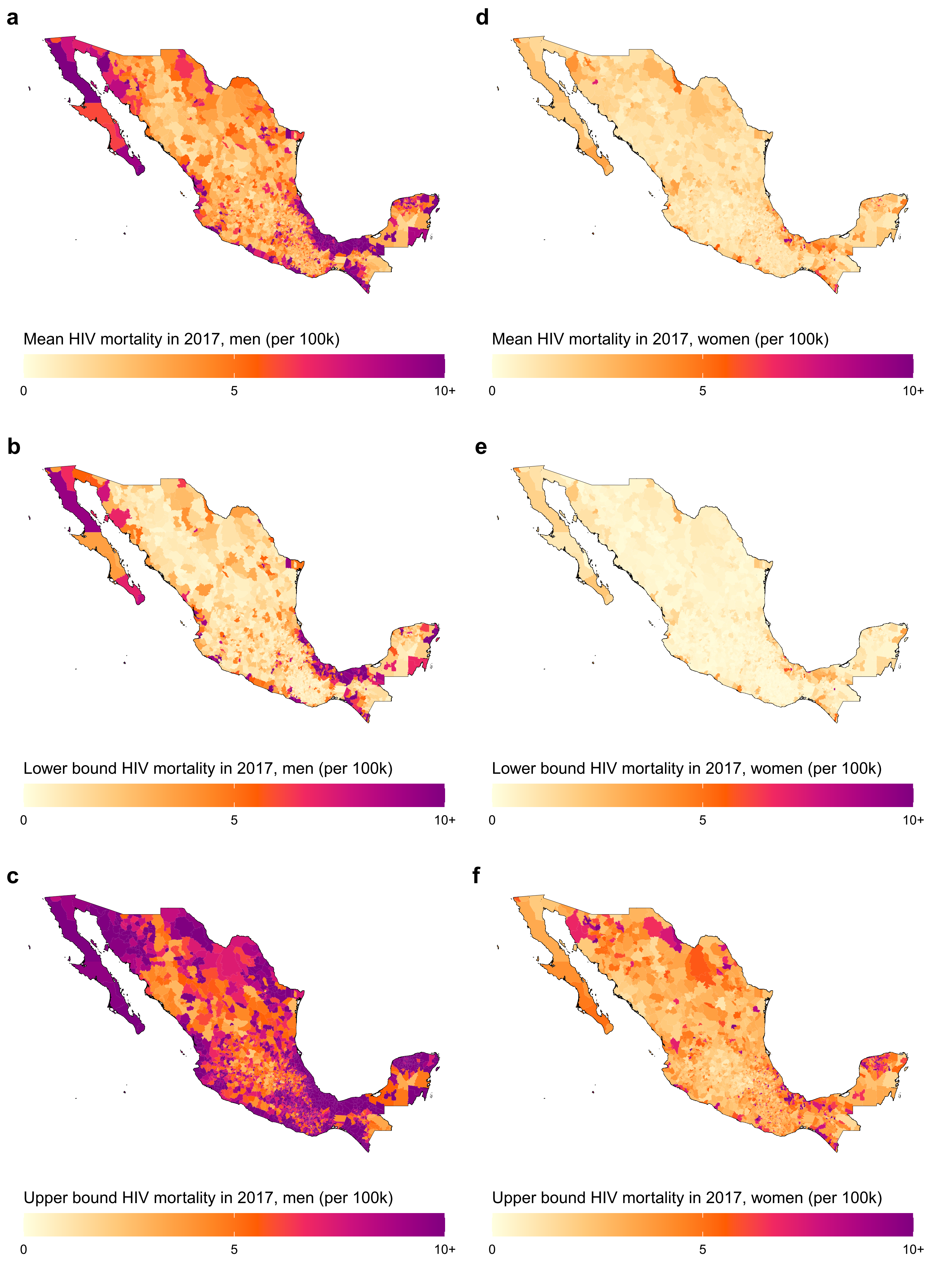


**Figure S15: Mean and uncertainty in estimated HIV mortality in Mexico, 2017.**  Estimated HIV mortality at the municipality level in 2017 in Mexico among men (**a-c**) and women (**d-f**). Mean estimates and lower and upper bounds of the 95% uncertainty intervals are shown in the top, middle, and bottom column, respectively.

## Figure S16: Estimated HIV mortality in Brazil by age group, 2017


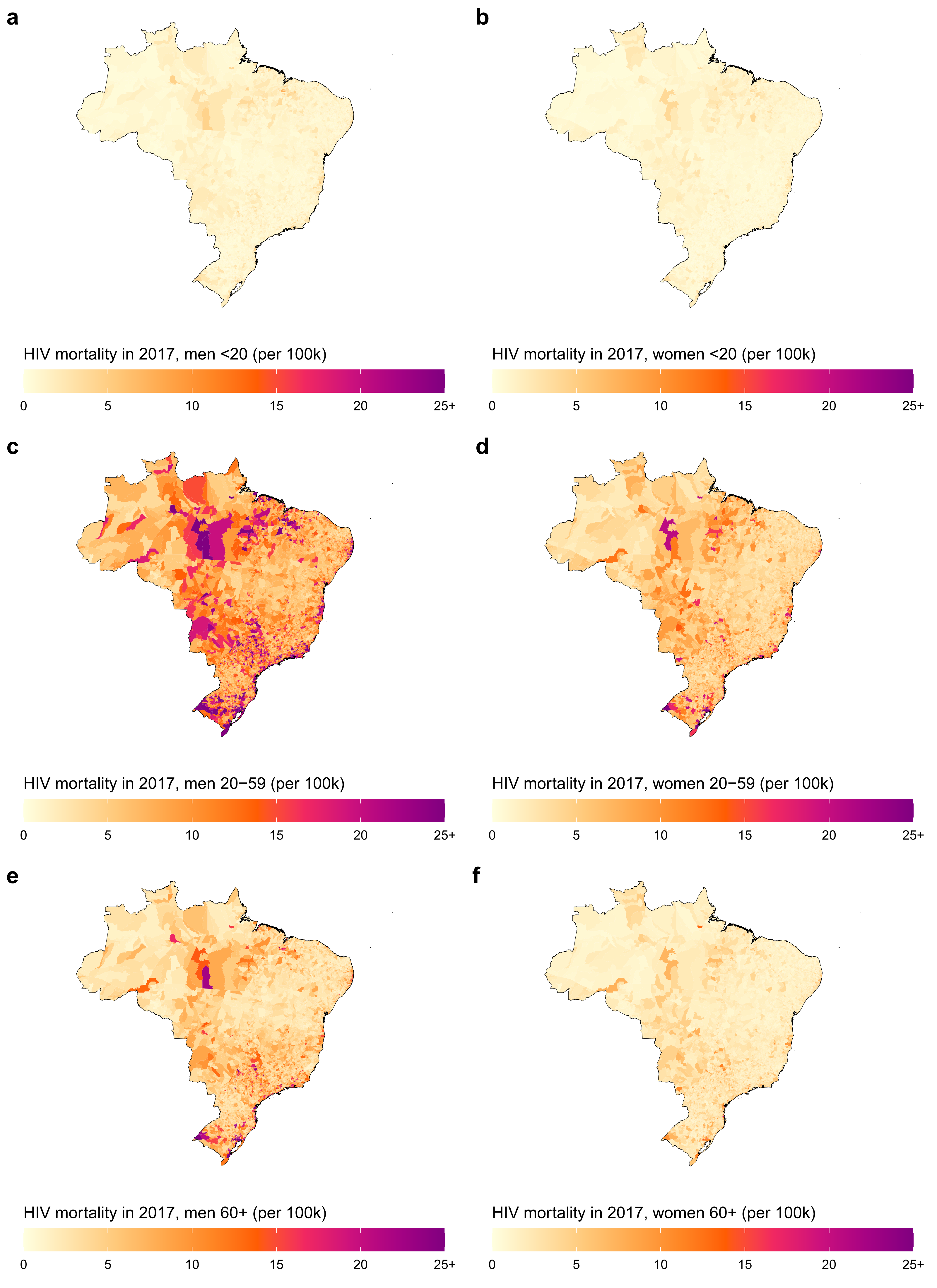


**Figure S16: Estimated HIV mortality in Brazil by age group, 2017.**  Estimated HIV mortality at the municipality level in 2017 in Brazil among men (**a**) and women (**b**) less than 20 years of age, among men (**c**) and women (**d**) between 20 and 59 years of age, and among men (**e**) and women (**f**) over 60 years of age.

## Figure S17: Estimated HIV mortality in Colombia by age group, 2017


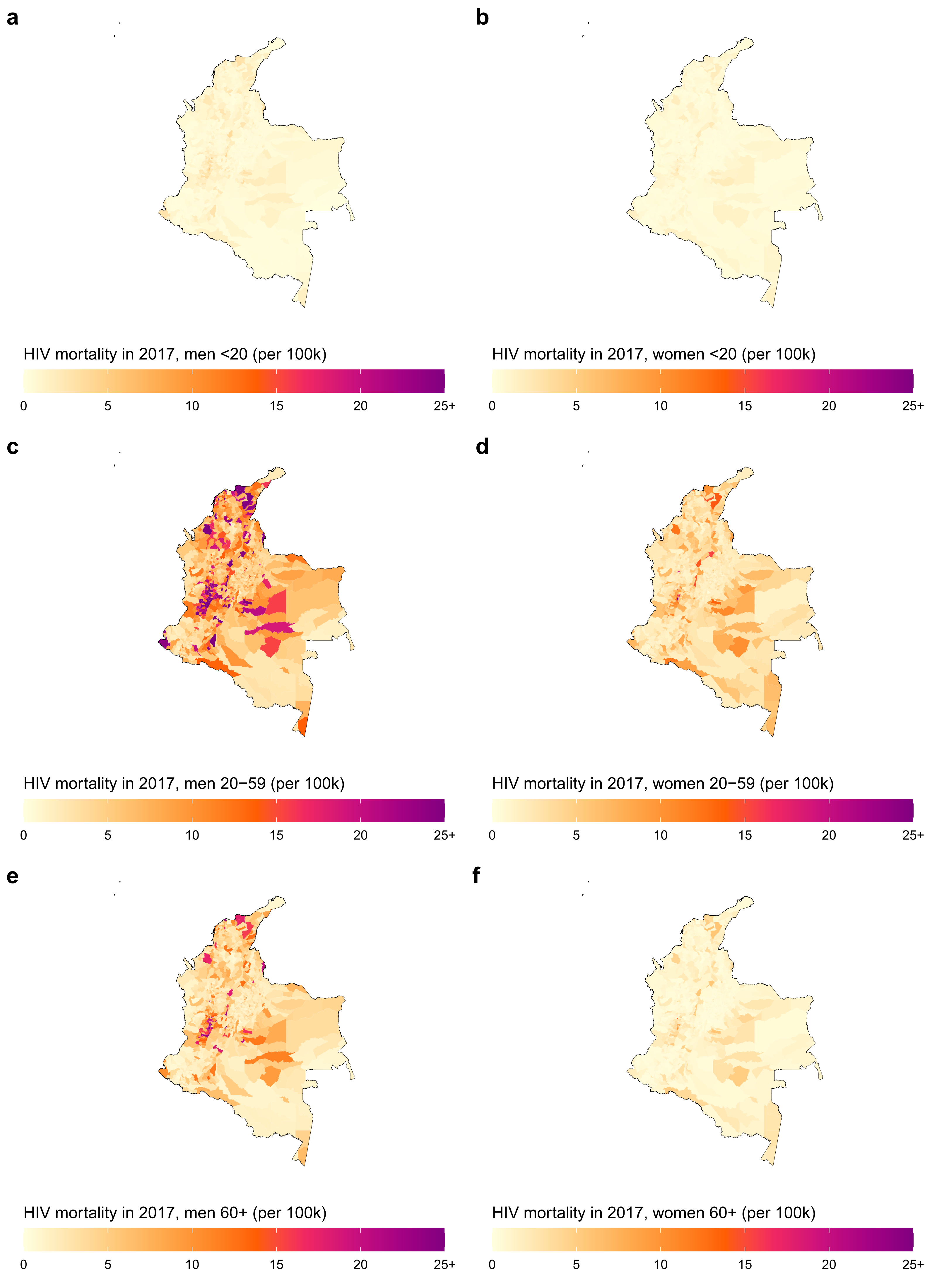


**Figure S17: Estimated HIV mortality in Colombia by age group, 2017.**  Estimated HIV mortality at the municipality level in 2017 in Colombia among men (**a**) and women (**b**) less than 20 years of age, among men (**c**) and women (**d**) between 20 and 59 years of age, and among men (**e**) and women (**f**) over 60 years of age.

## Figure S18: Estimated HIV mortality in Costa Rica by age group, 2016


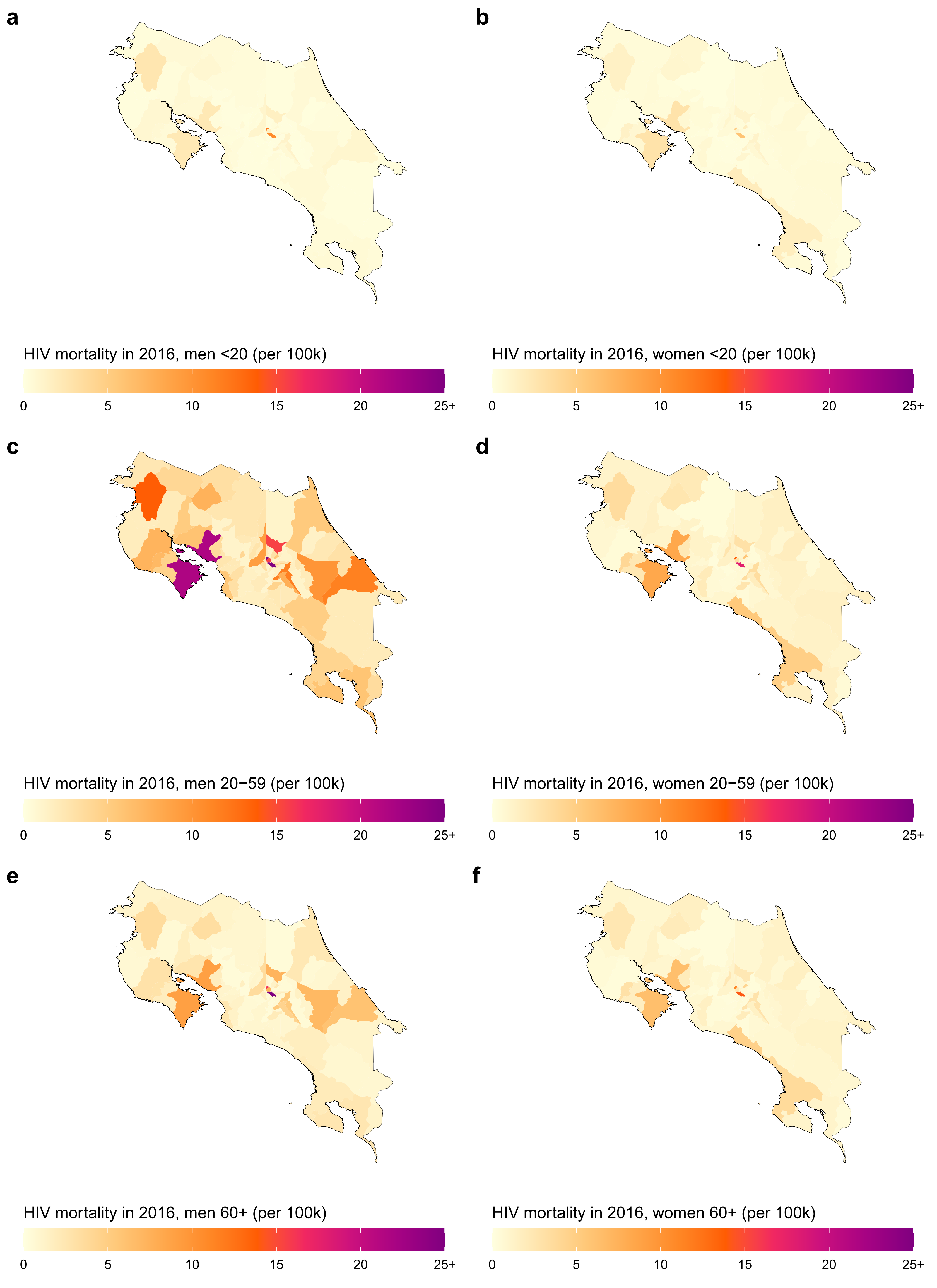


**Figure S18: Estimated HIV mortality in Costa Rica by age group, 2016.**  Estimated HIV mortality at the canton level in 2016 in Costa Rica among men (**a**) and women (**b**) less than 20 years of age, among men (**c**) and women (**d**) between 20 and 59 years of age, and among men (**e**) and women (**f**) over 60 years of age.

## Figure S19: Estimated HIV mortality in Ecuador by age group, 2014


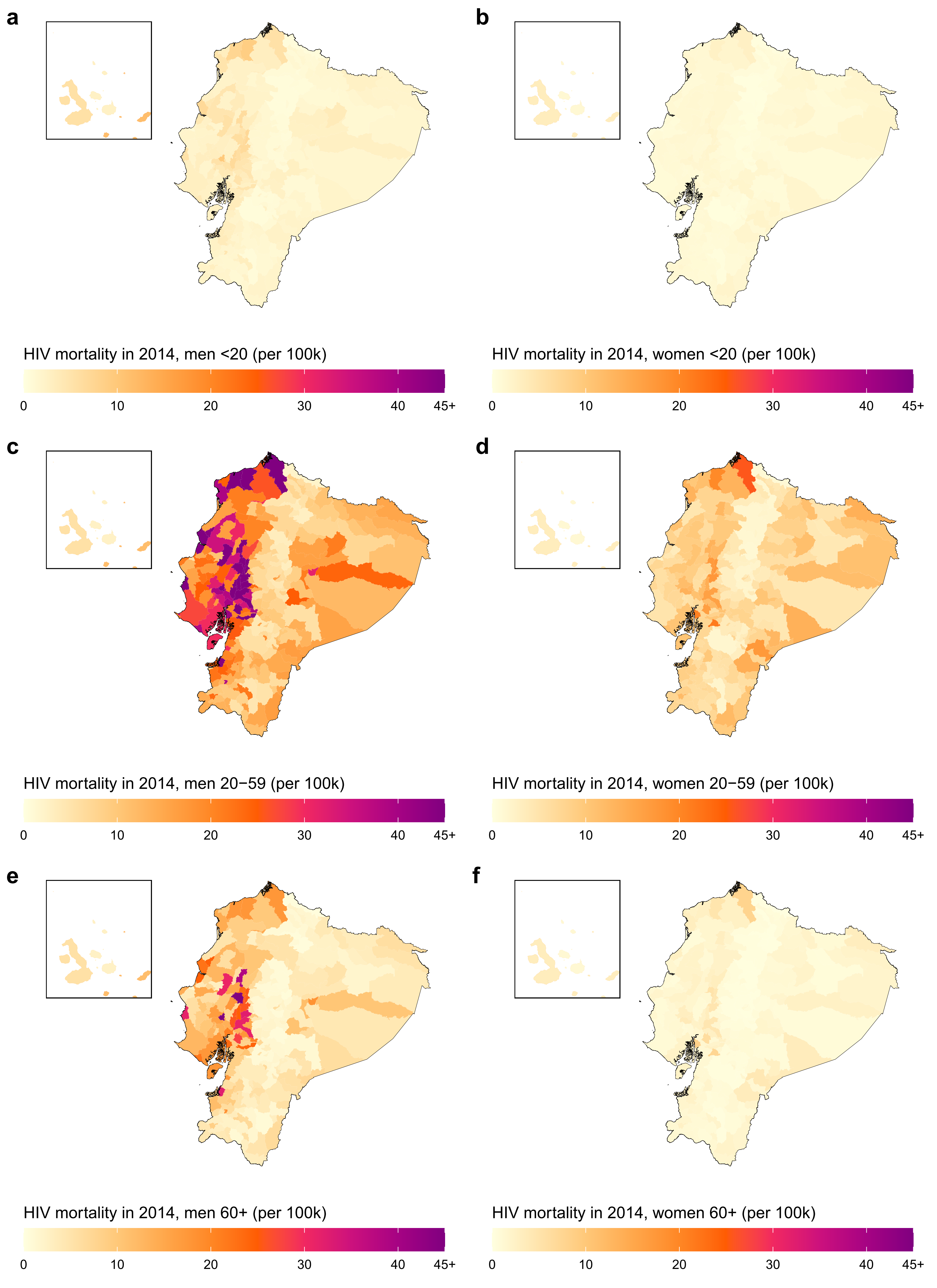


**Figure S19: Estimated HIV mortality in Ecuador by age group, 2014.**  Estimated HIV mortality at the canton level in 2014 in Ecuador among men (**a**) and women (**b**) less than 20 years of age, among men (**c**) and women (**d**) between 20 and 59 years of age, and among men (**e**) and women (**f**) over 60 years of age.

## Figure S20: Estimated HIV mortality in Guatemala by age group, 2017


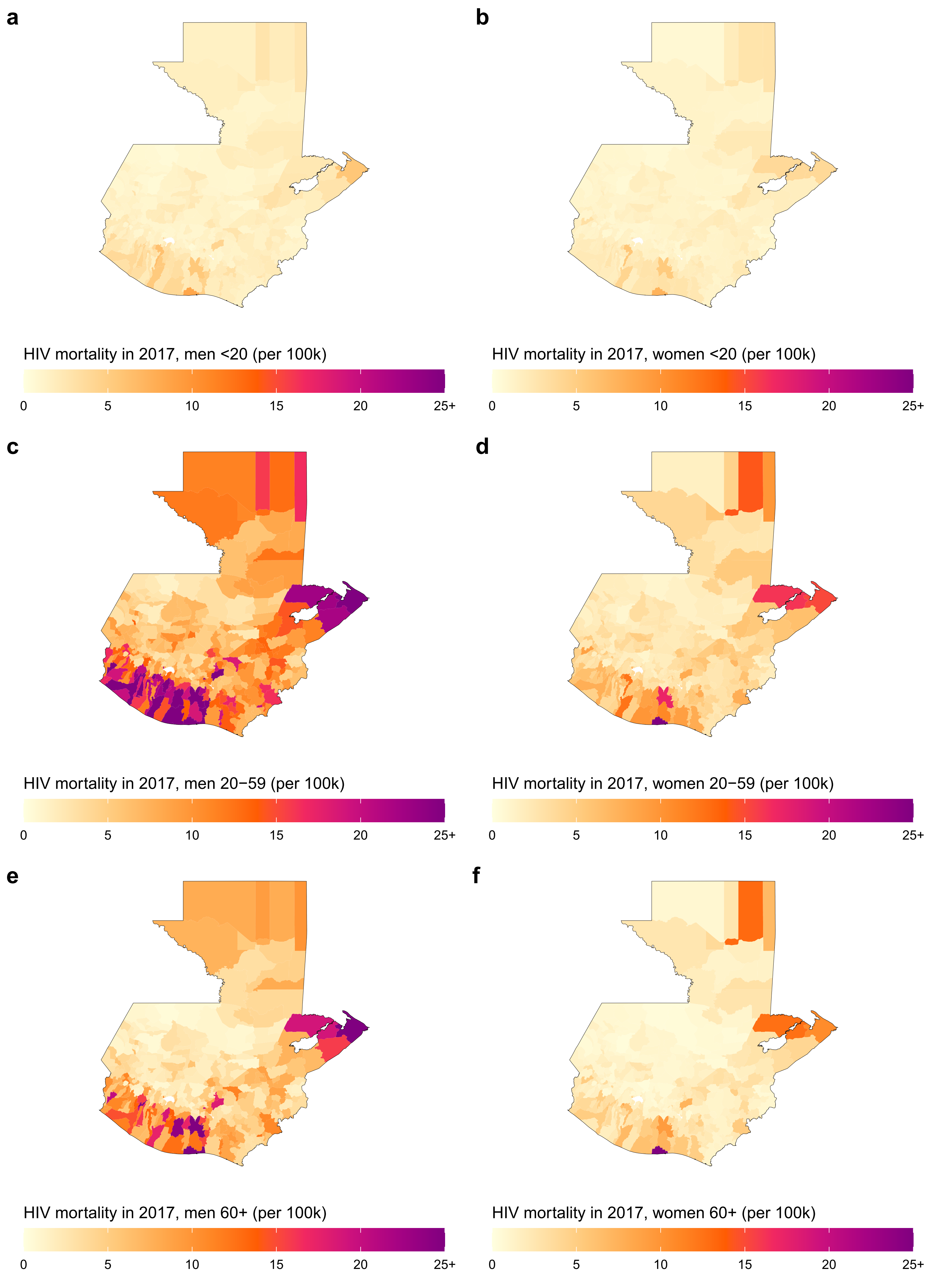


**Figure S20: Estimated HIV mortality in Guatemala by age group, 2017.**  Estimated HIV mortality at the municipality level in 2017 in Guatemala among men (**a**) and women (**b**) less than 20 years of age, among men (**c**) and women (**d**) between 20 and 59 years of age, and among men (**e**) and women (**f**) over 60 years of age.

## Figure S21: Estimated HIV mortality in Mexico by age group, 2017


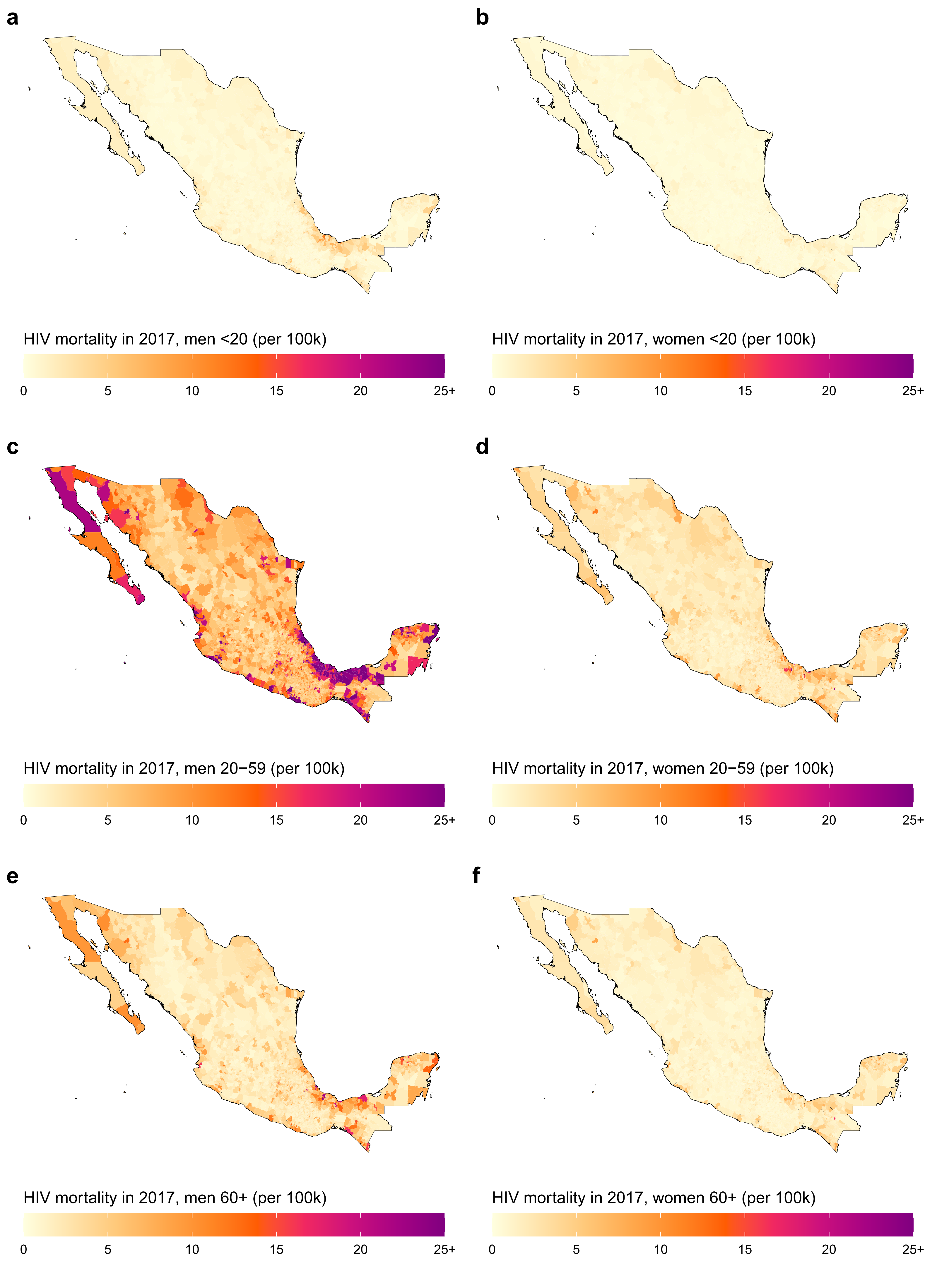


**Figure S21: Estimated HIV mortality in Mexico by age group, 2017.**  Estimated HIV mortality at the municipality level in 2017 in Mexico among men (**a**) and women (**b**) less than 20 years of age, among men (**c**) and women (**d**) between 20 and 59 years of age, and among men (**e**) and women (**f**) over 60 years of age.

# Supplemental tables

## Table S1: Merged municipalities by country to form stable geographical units

| **Country** | **State** | **Group** | **Areas** |
| --- | --- | --- | --- |
| Brazil | Para | 1 | Mojui dos Campos, Santarem |
| Brazil | Piaui | 1 | Altos, Pau D'Arco do Piaui |
| Brazil | Piaui | 2 | Aroeiras do Itaim, Picos |
| Brazil | Piaui | 3 | Nazaria, Teresina |
| Brazil | Rio Grande do Norte | 1 | Jundia, Varzea |
| Brazil | Alagoas | 1 | Coruripe, Jequia da Praia, Sao Miguel dos Campos |
| Brazil | Bahia | 1 | Barreiras, Luis Eduardo Magalhaes |
| Brazil | Bahia | 2 | Barrocas, Serrinha |
| Brazil | Espirito Santo | 1 | Colatina, Governador Lindenberg |
| Brazil | Rio de Janeiro | 1 | Mesquita, Nova Iguacu |
| Brazil | Santa Catarina | 1 | Criciuma, Balneario Rincao |
| Brazil | Santa Catarina | 2 | Laguna, Pescaria Brava |
| Brazil | Rio Grande do Sul | 1 | Acegua, Bage |
| Brazil | Rio Grande do Sul | 2 | Agua Santa, Caseiros, Ibiaca, Santa Cecilia do Sul, Tapejara |
| Brazil | Rio Grande do Sul | 3 | Almirante Tamandare do Sul, Carazinho |
| Brazil | Rio Grande do Sul | 4 | Arroio do Padre, Pelotas |
| Brazil | Rio Grande do Sul | 5 | Augusto Pestana, Boa Vista do Cadeado, Boa Vista do Incra, Bozano, Cruz Alta, Fortaleza dos Valos, Ijui |
| Brazil | Rio Grande do Sul | 6 | Barao de Cotegipe, Erechim, Jacutinga, Paulo Bento, Ponte Preta, Quatro Irmaos |
| Brazil | Rio Grande do Sul | 7 | Bento Goncalves, Pinto Bandeira, Pinto Bandeira |
| Brazil | Rio Grande do Sul | 8 | Caibate, Mato Queimado |
| Brazil | Rio Grande do Sul | 9 | Campinas do Sul, Cruzaltense |
| Brazil | Rio Grande do Sul | 10 | Canudos do Vale, Forquetinha, Lajeado, Progresso |
| Brazil | Rio Grande do Sul | 11 | Capao Bonito do Sul, Lagoa Vermelha |
| Brazil | Rio Grande do Sul | 12 | Capao do Cipo, Santiago, Sao Miguel das Missoes |
| Brazil | Rio Grande do Sul | 13 | Constantina, Novo Xingu |
| Brazil | Rio Grande do Sul | 14 | Coqueiro Baixo, Nova Brescia, Relvado |
| Brazil | Rio Grande do Sul | 15 | Coronel Pilar, Garibaldi, Roca Sales |
| Brazil | Rio Grande do Sul | 16 | Ernestina, Ibirapuita, Tio Hugo, Victor Graeff |
| Brazil | Rio Grande do Sul | 17 | Herval, Pedras Altas, Pinheiro Machado |
| Brazil | Rio Grande do Sul | 18 | Esmeralda, Pinhal da Serra |
| Brazil | Rio Grande do Sul | 19 | Espumoso, Jacuizinho, Salto do Jacui |
| Brazil | Rio Grande do Sul | 20 | Imigrante, Teutonia, Westfalia |
| Brazil | Rio Grande do Sul | 21 | Itati, Terra de Areia |
| Brazil | Rio Grande do Sul | 22 | Lagoa Bonita do Sul, Sobradinho |
| Brazil | Rio Grande do Sul | 23 | Marata, Montenegro, Salvador do Sul, Sao José do Sul |
| Brazil | Rio Grande do Sul | 24 | Palmeira das Missoes, Sao Pedro das Missoes |
| Brazil | Rio Grande do Sul | 25 | Rolador, Sao Luiz Gonzaga |
| Brazil | Rio Grande do Sul | 26 | Santa Margarida do Sul, Sao Gabriel |
| Brazil | Mato Grosso do Sul | 1 | Agua Clara, Camapua, Chapadao do Sul, Costa Rica, Figueirao, Paraiso das Aguas |
| Brazil | Mato Grosso | 1 | Agua Boa, Nova Nazare |
| Brazil | Mato Grosso | 2 | Alto Boa Vista, Bom Jesus do Araguaia, Cocalinho, Novo Santo Antonio, Ribeirao Cascalheira, Sao Felix do Araguaia, Serra Nova Dourada |
| Brazil | Mato Grosso | 3 | Aripuana, Colniza, Rondolandia |
| Brazil | Mato Grosso | 4 | Caceres, Curvelandia, Lambari D'Oeste, Mirassol d'Oeste |
| Brazil | Mato Grosso | 5 | Claudia, Itauba, Nova Santa Helena |
| Brazil | Mato Grosso | 6 | Conquista D'Oeste, Pontes e Lacerda, Vale de Sao Domingos |
| Brazil | Mato Grosso | 7 | Ipiranga do Norte, Itanhanga, Tapurah |
| Brazil | Mato Grosso | 8 | Nova Mutum, Santa Rita do Trivelato |
| Brazil | Mato Grosso | 9 | Novo Sao Joaquim, Santo Antonio do Leste |
| Brazil | Mato Grosso | 10 | Sao José do Xingu, Santa Cruz do Xingu |
| Brazil | Goias | 1 | Anapolis, Campo Limpo de Goias |
| Brazil | Goias | 2 | Ceres, Ipiranga de Goias |
| Brazil | Goias | 3 | Gameleira de Goias, Silvania |
| Brazil | Goias | 4 | Itaja, Lagoa Santa |
| Colombia | Bolivar | 1 | Norosi, Rio Viejo |
| Colombia | Cauca | 1 | Caloto, Guachene |
| Colombia | Cordoba | 1 | Montelibano, San José De Ure |
| Colombia | Cordoba | 2 | San Andres Sotavento, Tuchin |
| Colombia | Sucre | 1 | Covenas, Santiago De Tolu |
| Colombia | Amazonas | 1 | El Encanto, Puerto Alegria |
| Colombia | Guainia | 1 | Barranco Minas, Mapiripana |
| Costa Rica | Puntarenas | 1 | Aguirre, Aguirre |
| Ecuador | Los Rios | 1 | Quinsaloma, Ventanas |
| Ecuador | Pichincha, Santo Domingo De Los Tsachilas | 1 | Santo Domingo, Santo Domingo |
| Ecuador | Esmeraldas, Santo Domingo De Los Tsachilas, Zona No Delimitada | 1 | La Concordia, La Independencia, Plan Piloto, Quininde |
| Ecuador | Guayas, Santa Elena | 1 | Santa Elena, Santa Elena |
| Ecuador | Guayas, Santa Elena | 2 | La Libertad, La Libertad |
| Ecuador | Guayas, Santa Elena | 3 | Salinas, Salinas |
| Guatemala | Suchitepequez | 1 | Cuyotenango, San José La Maquina |
| Guatemala | San Marcos | 1 | La Blanca, Ocos |
| Guatemala | Huehuetenango | 1 | Concepcion Huista, Petatan |
| Guatemala | El Peten | 1 | La Libertad, Las Cruces |
| Guatemala | El Peten | 2 | Dolores, El Chal |
| Guatemala | Zacapa | 1 | San Jorge, Zacapa |
| Guatemala | Escuintla | 1 | La Gomera, Sipacate |
| Mexico | Guerrero | 1 | Azoyu, Cuajinicuilapa, Juchitan, Marquelia |
| Mexico | Guerrero | 2 | Chilapa de Alvarez, José Joaquin de Herrera |
| Mexico | Guerrero | 3 | Iliatenco, Malinaltepec, San Luis Acatlan |
| Mexico | Guerrero | 4 | Cochoapa el Grande, Metlatonoc |
| Mexico | Jalisco | 1 | Arandas, San Ignacio Cerro Gordo |
| Mexico | Mexico | 1 | Jaltenco, Tonanitla |
| Mexico | Mexico | 2 | San Felipe del Progreso, San José del Rincon |
| Mexico | Mexico | 3 | Luvianos, Tejupilco |
| Mexico | Quintana Roo | 1 | Bacalar, Othon P. Blanco |
| Mexico | Quintana Roo | 2 | Benito Juarez, Puerto Morelos |
| Mexico | Quintana Roo | 3 | Solidaridad, Tulum |
| Mexico | Veracruz de Ignacio de la Llave | 1 | Martinez de la Torre, San Rafael |
| Mexico | Veracruz de Ignacio de la Llave | 2 | Playa Vicente, Santiago Sochiapan |
| Mexico | Zacatecas | 1 | Santa Maria de la Paz, Teul de Gonzalez Ortega |
| ***Group number differentiates between multiple merged areas that are within the same first-administrative level unit | | | |

## Table S2: Vital Registration data

| **Country** | **Years** | **Reference** | **NID^*^** |
| --- | --- | --- | --- |
| Brazil | 2000–2017 | Ministry of Health (Brazil). Brazil Mortality Information System – Deaths 2000-2017.Brasilia, Brazil: Ministry of Health (Brazil). | 153037, 153038,  153039, 153040,  153041, 153042,  153043, 153044,  153045, 153046,  153048, 153049,  153050, 173779,  265226, 268267,  317728, 386735 |
| Colombia | 2000–2017 | National Administrative Department of Statistics (DANE) (Colombia). Colombia Vital Statistics - Deaths 2000-2017. Bogotá, Colombia: National Administrative Department of Statistics (DANE) (Colombia). | 397407, 397409, 397411, 397413,  397415, 397417,  397419, 397421,  65267, 65199,  57982, 265177,  265178, 265179,  265181, 265219,  265220, 396797 |
| Costa Rica | 2014– 2016 | National Institute of Statistics and Censuses (Costa Rica). Costa Rica Registered Deaths 2014-2016. San José, Costa Rica: National Institute of Statistics and Censuses (Costa Rica). | 398942, 325066, 398943 |
| Ecuador | 2004– 2014 | National Institute of Statistics and Censuses (Ecuador). Ecuador General Deaths 2004-2014. Quito, Ecuador: National Institute of Statistics and Censuses (Ecuador). | 343283, 343285,  343287, 343289,  256676, 256677,  256678, 256679,  256680, 256681,  325080 |
| Guatemala | 2009–  2017 | National Statistics Institute (Guatemala). Guatemala Vital Statistics 2009-2017. Guatemala City, Guatemala: National Statistics Institute (Guatemala). | 336252, 336251,  336250, 240728,  240729, 240730,  286175, 335901,  398900 |
| Mexico | 2000–2017 | National Institute of Statistics and Geography (INEGI) (Mexico). Mexico Vital Registration - Deaths 2000-2016. | 116138, 116139,  116140, 116141,  116142, 116143,  116144, 116145,  116146, 93775,  116147, 107113,  124425, 157617,  240409, 281783,  325345, 386753 |

*NID = Data source unique identifier in the Global Health Data Exchange (http://ghdx.healthdata.org/). Additional information about each data sources is available via the GHDx, including information about the data provider and links to where the data can be accessed or requested (where available). NIDs can be entered in the search bar to retrieve the record for a particular source.

## Table S3: Covariate data sources

| **Covariate** | **Temporal resolution** | **Source** | **Reference** | **NID^*^** |
| --- | --- | --- | --- | --- |
| Population Density | 2000–2017 | WorldPop | Geography and Environmental Science, University of Southampton, WorldPop. Age and Sex Structures, Global Per Country 2000-2020. Southampton, United Kingdom: WorldPop, 2018. | 420764 |
| Nighttime lights | 2000–2013 | NOAA DMSP | National Oceanic and Atmospheric Administration (NOAA) (United States), United States Air Force (USAF). DMSP-OLS Nighttime Lights Time Series, V4. United States of America: National Oceanic and Atmospheric Administration (NOAA) (United States). | 418630 |
| Travel time to the nearest settlement > 50,000 inhabitants | None | Malaria Atlas Project, Big Data Institute, Nuffield Department of Medicine, University of Oxford | Nelson A, Joint Research Centre of the European Commission. Estimated travel time to the nearest city of 50,000 or more people in year 2000. Ispra, Italy: Global Environment Monitoring Unit, Joint Research Centre of the European Commission, 2008. | 316680 |
| Urbanicity | 2000–2015 | European Commission/ GHS | Pesaresi, Martino; Freire, Sergio (2016): GHS settlement grid, following the REGIO model 2014 in application to GHSL Landsat and CIESIN GPW v4-multitemporal (1975-1990-2000-2015). European Commission, Joint Research Centre (JRC) [Dataset] PID: http://data.europa.eu/89h/jrc-ghsl-ghs_smod_pop_globe_r2016a | 418851 |

*NID = Data source unique identifier in the Global Health Data Exchange (http://ghdx.healthdata.org/). Additional information about each data sources is available via the GHDx, including information about the data provider and links to where the data can be accessed or requested (where available). NIDs can be entered in the search bar to retrieve the record for a particular source.

## Table S4: National HIV mortality rates among men and women

| **HIV/AIDS mortality (age-standardized rates per 100,000) among men and women in six Latin American countries in the first and latest year of study** | | | | | | |
| --- | --- | --- | --- | --- | --- | --- |
| Country | HIV mortality rate, (95% UI)^*^ | | | | | |
|  | Men | | Women | | Both Sexes | |
|  | First Year | Latest Year | First Year | Latest Year | First Year | Latest Year |
| Brazil | 11.7 (11.4-11.9) | 8.7 (8.5-8.9) | 5.3 (5.1-5.4) | 4.5 (4.4-4.6) | 8.4 (8.2-8.6) | 6.5 (6.4-6.7) |
| Colombia | 9.8 (9.3-10.2) | 7.8 (7.5-8.1) | 2.1 (1.9-2.3) | 2.5 (2.3-2.7) | 5.8 (5.5-6.1) | 5.0 (4.8-5.3) |
| Costa Rica | 6 (5.1-7.1) | 4.9 (4.2-5.8) | 1.9 (1.4-2.7) | 1.5 (1.0-2.3) | 3.9 (3.2-4.7) | 3.2 (2.5-3.9) |
| Ecuador | 8.5 (7.8-9.2) | 10.9 (10.1-11.7) | 2.1 (1.8-2.4) | 3.4 (3-3.8) | 5.2 (4.7-5.7) | 7.0 (6.5-7.6) |
| Guatemala | 10.8 (9.9-11.7) | 6.8 (6.2-7.4) | 4.1 (3.7-4.6) | 2.8 (2.4-3.1) | 7.2 (6.5-7.9) | 4.6 (4.1-5.1) |
| Mexico | 9 (8.8-9.3) | 6.9 (6.7-7.1) | 2.0 (1.9-2.1) | 1.9 (1.8-2) | 5.4 (5.2-5.5) | 4.3 (4.1-4.4) |

^*^UI = 95% uncertainty intervals
